# Non-Pharmaceutical Interventions against COVID-19 Pandemic: Review of Contact Tracing and Social Distancing Technologies, Protocols, Apps, Security and Open Research Directions

**DOI:** 10.3390/s22010280

**Published:** 2021-12-30

**Authors:** Uzoma Rita Alo, Friday Onwe Nkwo, Henry Friday Nweke, Ifeanyi Isaiah Achi, Henry Anayo Okemiri

**Affiliations:** 1 Department of Computer Science and Informatics, Alex Ekwueme Federal University, Ndufu-Alike, Ikwo P.M.B 1010, Abakaliki 480211, Ebonyi State, Nigeria; friday.nkwo@funai.edu.ng (F.O.N.); achi.ifeanyi@funai.edu.ng (I.I.A.); henry.okemiri@funai.edu.ng (H.A.O.); 2 Centre for Research in Machine Learning, Artificial Intelligence and Network Systems, Computer Science Department, Ebonyi State University, P.M.B 053, Abakaliki 480211, Ebonyi State, Nigeria; henry.nweke@ebsu.edu.ng

**Keywords:** COVID-19, sensor technologies, contact tracing, social distancing, internet of things, artificial intelligence, review

## Abstract

The COVID-19 Pandemic has punched a devastating blow on the majority of the world’s population. Millions of people have been infected while hundreds of thousands have died of the disease throwing many families into mourning and other psychological torments. It has also crippled the economy of many countries of the world leading to job losses, high inflation, and dwindling Gross Domestic Product (GDP). The duo of social distancing and contact tracing are the major technological-based non-pharmaceutical public health intervention strategies adopted for combating the dreaded disease. These technologies have been deployed by different countries around the world to achieve effective and efficient means of maintaining appropriate distance and tracking the transmission pattern of the diseases or identifying those at high risk of infecting others. This paper aims to synthesize the research efforts on contact tracing and social distancing to minimize the spread of COVID-19. The paper critically and comprehensively reviews contact tracing technologies, protocols, and mobile applications (apps) that were recently developed and deployed against the coronavirus disease. Furthermore, the paper discusses social distancing technologies, appropriate methods to maintain distances, regulations, isolation/quarantine, and interaction strategies. In addition, the paper highlights different security/privacy vulnerabilities identified in contact tracing and social distancing technologies and solutions against these vulnerabilities. We also x-rayed the strengths and weaknesses of the various technologies concerning their application in contact tracing and social distancing. Finally, the paper proposed insightful recommendations and open research directions in contact tracing and social distancing that could assist researchers, developers, and governments in implementing new technological methods to combat the menace of COVID-19.

## 1. Introduction

Since 30 January 2020, when the World Health Organization (WHO) announced the outbreak of COVID-19, the global stories have been pathetic. People have been dying in pitiable numbers, pushing their dependents into misery, untold hardship, and unexplainable difficulties. Furthermore, businesses have been shut down and human movements are restricted as a measure to curtail the disease. The impact has been devastating on the economy as many organizations have had their businesses closed or partially in operation. Consequently, the outbreak has led to staff retrenchments, loss of revenues, and dwindling gross domestic products (GDP) in most economies around the world. It is known that infectious disease outbreaks such as the COVID-19 are usually characterized by a lack of immediate vaccines and cures. As a result, infection control measures such as contact tracing and social distancing are usually employed by health authorities pending when vaccines and permanent cures of such diseases are found [1].

Contact tracing is a public health intervention scheme aimed at timely identification, isolation, and diagnosis of people who are at the risk of contracting infectious diseases as a result of their close interactions with already infected persons. This scheme is necessary because some infected persons are healthy carriers who may not go down with the signs and symptoms of the disease but keep infecting others when they interact with them. The overall purpose of the contact tracing scheme is to truncate the infection path to halt the further spread of the disease [2]. Conventionally, contact tracing is performed through manual methods or technological-based methods. The manual method is performed by interviewing infected persons to get the names, addresses, and other details of people who came in close contact with them within a specified period believed to be the incubation period of the infectious disease. The named contacts are thereafter invited for diagnosis and interviews repeating the cycle until the infectious disease is fully contained. However, the use of manual approaches for contacting tracing has been observed to be tedious, expensive, and time-consuming. Moreover, the effectiveness of the manual approach cannot be guaranteed since the infected persons have to solely rely on the recollection ability of the fallible human memory for contact data collection. Another limiting factor is that the privacy of both the infected persons and their contacts cannot be protected in the manual scheme [3,4]. Due to the inefficiency of manual-based contact tracing methods, technological-based approaches are widely adopted to minimize the spread of coronavirus disease. The technological approaches of contact processing are the main focus of this paper.

On the other hand, social distancing is a public health intervention scheme aimed at minimizing close human interactions and physical contact that may lead to the spread of deadly diseases [5]. For effective social distancing against COVID-19, it has been recommended that people should keep a minimum of two meters distance from each other [6]. Traditionally, social distancing is implemented using policy statements by government authorities such as the closure of public places, placing a ban on public gatherings and events such as funerals, wedding ceremonies, etc. [7]. However, these state-of-the-art approaches not only cause discomfort to the people but also negatively impact their social life and source of livelihood [8,9,10].

In this context, studies have proposed various technology-based methods to automatically detect individuals that might have come in contact with COVID-19 infected persons or are at risk of spreading the disease. Similarly, government agencies and institutions have proposed different social distancing mechanisms using technologies to minimize the spread of COVID-19 by reducing the frequency of closeness [2,5]. These technologies have played vital roles in both contact tracing and social distancing enforcement. Some of these technologies include smartphone devices and their embedded technologies such as Bluetooth low energy, Wi-Fi, RFID, and magnetometer sensors. Others include Blockchain, artificial intelligence, and computer vision methods [11,12]. This insightful paper discusses contact tracing and social distancing technologies against COVID-19.

**Figure 1:**
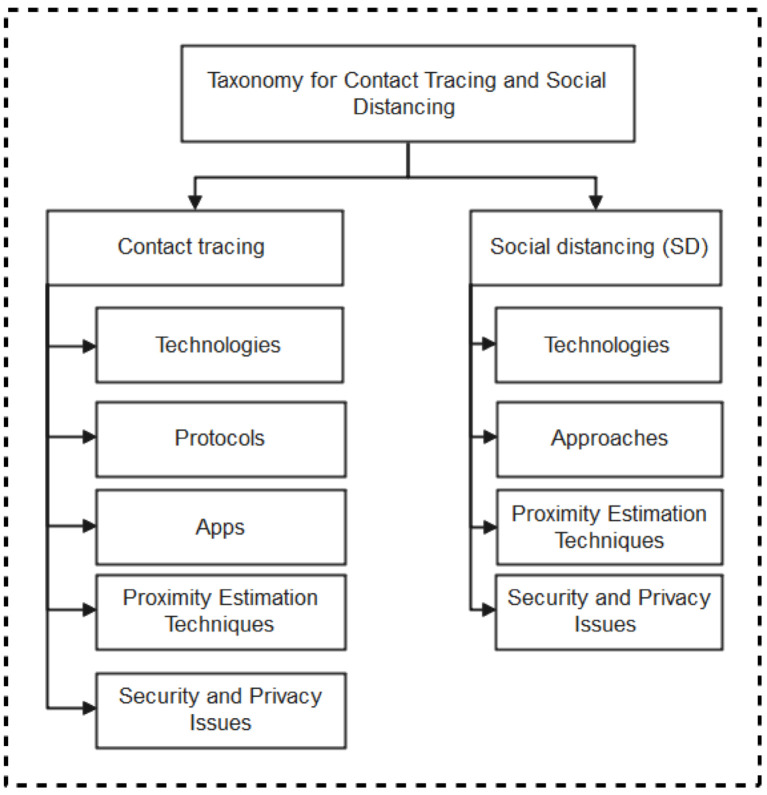
Taxonomy of contact tracing and social distancing methods to combat Covid-19.

As summarized in the taxonomy shown in Figure 1, the paper highlights contact tracing technologies, recent protocols, apps, and security/vulnerabilities inherent in recently proposed literature. In addition, we outlined the strengths and weaknesses of each protocol to enable the research community to make an informed decision while developing contact tracing intervention strategies against COVID-19. Moreover, the paper provides an all-inclusive survey of social distancing, distance measures, automated crowd control, face mask detection, isolation/quarantine, and how to maintain social interaction in the COVID-19 period through virtual means.

Most of the existing literature on non-pharmaceutical intervention strategies against coronavirus disease separately focused on contact tracing and social distancing. Some studies have either focused on contact tracing [12,13,14] or social distancing techniques [11,15] to minimize the spread of coronavirus. To the best of our knowledge, none of the existing studies have comprehensively surveyed the feasibility of deploying both contact tracing and social distancing approaches as non-pharmaceutical strategies against COVID-19. Table 1 highlights some of the existing studies and their limitations. From this table, we can conclude that most of the existing reviews have failed to take into consideration recent technologies such as artificial intelligence, cellular networks, Blockchain, proximity estimation methods, etc. in developing efficient contact tracing and social distancing apps. Additionally, the strengths and weaknesses of various protocols recently developed by different researchers were not highlighted in previous studies. These strengths and weaknesses would aid developers to make informed choices on the protocols to adopt. Moreover, from available studies in literature, there are no comprehensive surveys or reviews that provide important discussions on the intersection of contact tracing and social distancing technologies, protocols, apps, and security/vulnerability as non-pharmaceutical intervention strategies to minimize the spread of the COVID-19 pandemic. The main goal of this paper is to guide technology-based intervention strategy researchers and developers in contact tracing and social distancing on the major technologies, protocols, methods, and apps, and future research prospects that require further focus.

### Contributions of the Study

This study provides an extensive review of contact tracing and social distancing technologies as non-pharmaceutical interventions against the COVID-19 pandemic. In summary, the contributions of this review to the current body of knowledge include:

- In-depth exploration of recent contact tracing and social distancing schemes highlighting their various strengths and weaknesses.
- More so, the study provides a comprehensive review of the technologies, protocols, apps, proximity estimation techniques, security and privacy issues in contact tracing.
- Furthermore, the study performs an in-debt analysis of the approaches, proximity estimation techniques, and security and privacy issues in social distancing.
- Finally, the study identifies and proposes some open research directions.

The remainder of the paper is organized as follows: Section 2 explains the various technologies employed in contact tracing highlighting the protocols and apps developed using each of the technologies. Section 3 presents various technologies and approaches for social distancing. In Section 4, the findings of the review are extensively discussed while in Section 5, open research directions were identified and proposed. The review is concluded in Section 6.

## 2. Technology-Based Contact Tracing Methods against COVID-19

Different technologies are being employed or proposed for deployment in contact tracing solutions against infectious diseases. Those technologies include proximity sensing technologies such as Bluetooth, Global Positioning System (GPS), and Artificial Intelligence (AI), and other technologies that possess high potential for implementation in contact tracing frameworks include Wi-Fi, Smartphone Magnetometer Traces, Cellular Networks, Near Field Communication (NFC), Radio Frequency Identifier (RFID), Blockchain, and Ultra-Wide Band. Many protocols have been proposed and apps developed leveraging these technologies [27]. In this subsection, these technologies are discussed highlighting their various features, protocols, and apps already deployed.

### 2.1. Bluetooth-Based Protocols and Apps for Contact Tracing

Bluetooth is a short-range communication protocol developed by a consortium of companies including Ericsson, IBM, Nokia, Toshiba, and many others who came together under the umbrella of Bluetooth special interest group (SIG) [28]. A newer version called Bluetooth low energy (BLE) was designed specifically for low energy applications such as in the internet of things (IoT) [29]. Consequently, BLEs have gained special attention in the development of contact tracing systems against COVID-19.

**Figure 2:**
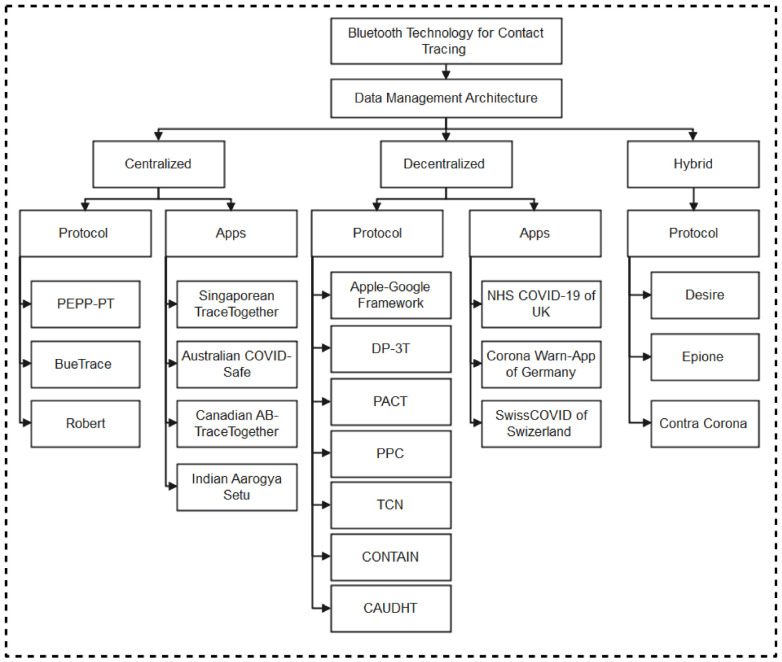
Bluetooth-based Protocols and Apps for contact tracing.

We, therefore, present protocols and applications developed using Bluetooth technology as shown in Figure 2. For clarity purposes, the classification of contact tracing protocols and apps are categorized into the three major data management methods: centralized, decentralized, and hybrid architectures [30].

#### 2.1.1. Centralized Architecture

**Figure 3:**
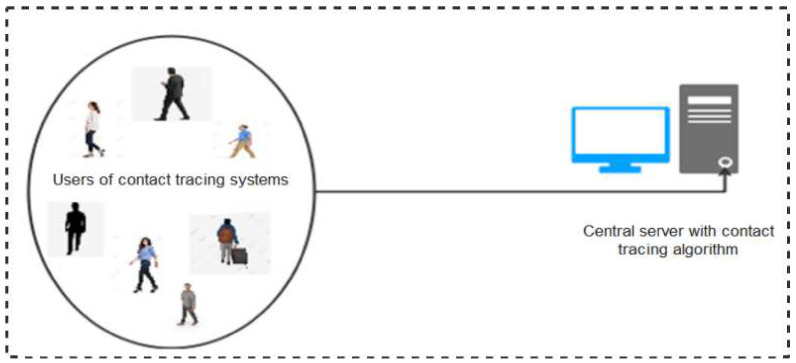
Conceptual diagram of centralized protocols.

The centralized protocol implies that user devices depend on a central server to perform key functions including computation of anonymous identifiers, data processing (reconstruction, encryption, and decryption), risk analysis, and sending of alerts to risky users informing them of their risk level. Under this architecture, users’ apps exchange anonymous Bluetooth identifiers (encrypted or/and randomized) and store them locally. When a user is infected, with the permission of a health authority, the stored data are uploaded to the central server. The central server thereafter performs risk-level computations and notifies risky users of their likely exposure to the disease. A schematic representation of the functionality of centralized protocols is shown in Figure 3. In this sub-section, we discuss the various Bluetooth-based protocols and apps developed leveraging on the centralized architectures.

**Pan-European Privacy-Preserving Proximity Tracing (PEPP-PT**): A foremost promoter of the centralized data management architecture in contact tracing systems is the Pan-European Privacy-Preserving Proximity Tracing (PEPP-PT), an international team consisting of more than 130 members across several European countries. The team is composed of a consortium of academics, technological experts, and business stakeholders whose aim is to provide a framework that will guide developers and countries in deploying effective and privacy-oriented contact tracing systems against the coronavirus. The framework is developed in full compliance with the European General Data Protection Regulation (GDPR) which implies that no personal or location information will be shared in the framework. Furthermore, the framework source code was made open and free [31]. In the PEPP-PT framework, the system assigns each user device a permanent Identification number (id) through which it creates pseudonyms broadcasted as Bluetooth IDs. The Bluetooth ids being broadcasted and sensed by other user devices are randomized pseudonyms to provide user privacy. Sensed data is stored in the user device’s local memory. Upon infection of a user, the infected user voluntarily uploads the stored data to a central server for risk computation and notification of the close contacts of infected persons.

However, PEPP-PT being a centralized framework suffers a single point of failure. This implies that any compromise or damage to the server will render the entire system useless. The PEPP-PT framework has also been accused of lack of transparency which led to the resignation of some of the team members [32].

**Figure 4:**
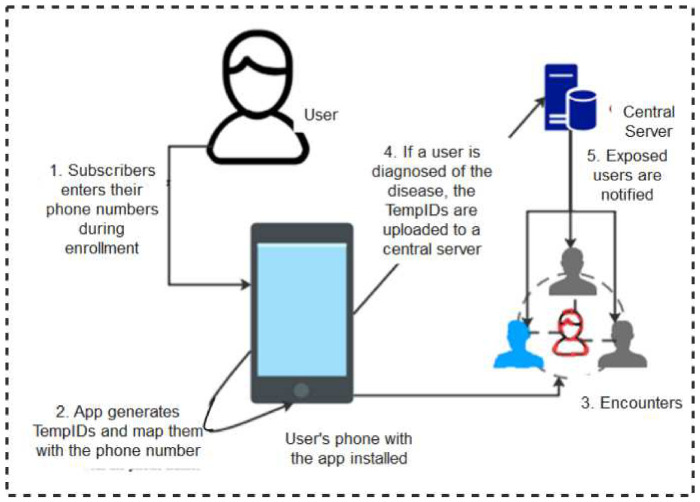
BlueTrace Protocol.

**Blue Trace:** The BlueTrace protocol [33] is powered by the Singaporean Government Digital Services. In this protocol, users’ phone numbers are mapped to the randomized temporary identities (TempIDs) generated for every subscriber. During encounters, user devices exchange TempIDs and store them locally in their local memories. If tested positive, the user uploads its contact details to a Health Authority server where the messages are decrypted and risky users contacted through their phone numbers. Figure 4 shows a schematic diagram of how the BlueTrace protocol works. The major stand-out area of this protocol is that the TempIDs are generated centrally and mapped to device phone numbers making it possible for the risky individuals to be identified and contacted without difficulty.

However, as applicable to every centralized system, the protocol suffers a single point of failure. This implies that any compromise or damage to the server where the TempIDs are stored will render the entire system useless. Furthermore, since the temps are mapped to devices’ phone numbers, an adversary may attack the system by sending fictitious messages to the phone numbers, hence creating panics capable of discrediting the system.

**Robust and Privacy-Preserving Proximity Tracing Protocol (ROBERT**): The Robust and Privacy-Preserving Proximity Tracing Protocol (ROBERT) [34] is powered by Inria and Fraunhofer which are French and German companies, respectively, and are members of the Pan-European Privacy-Preserving Proximity Tracing (PEPP-PT) project. The protocol adopts a centralized data structure in its privacy-preserving contact tracing solution. To enroll in the system, users download the apps and install them on their mobile devices. For every subscriber, unlike in BlueTrace, a permanent ID is assigned by the server with which it identifies the user. Each device creates some ephemeral Bluetooth IDs which are functions of the assigned permanent ID. During daily interactions with other users, the ephemeral Bluetooth IDs are exchanged and stored in the mobile devices of the users. If a user is diagnosed with coronavirus, the stored ephemeral Bluetooth IDs are voluntarily uploaded to the central server where the risk computation is performed and risky users notified. Similarly, this protocol also suffers a single point of failure since any compromise or damage to the central server implies that the entire system has failed.

#### 2.1.2. Decentralized Protocols

**Figure 5:**
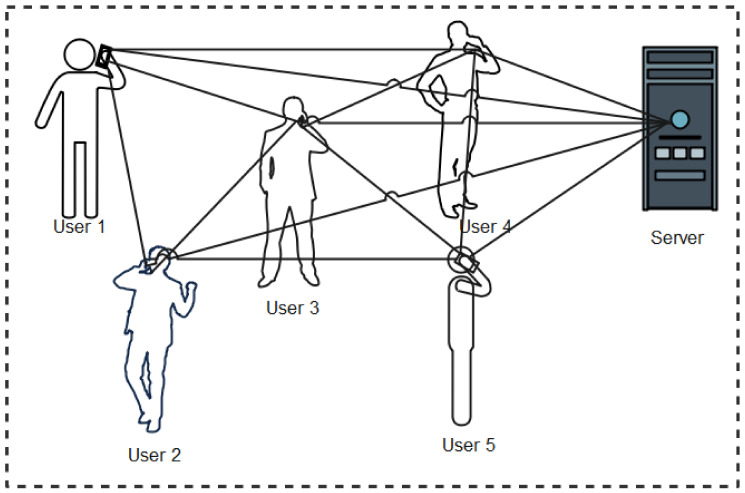
Conceptual diagram of decentralized contact tracing systems.

Moreover, in the decentralized protocols, user devices generate random ephemeral identifiers (EphID), beacons, or private seeds through which random anonymous keys (pseudonyms) are computed anonymous BLE IDs and stored locally in user devices. The EphIDs, beacon, or Pseudonyms are broadcasted as Bluetooth identifiers for nearby user devices to interact with and store as IDs of close contacts. Where necessary, the stored data is forwarded to a central system that carries out the decryption of the anonymous BLE IDs and provides the platform for other users to ascertain their risk level. When a user tests positive to the disease, they upload their stored EphIDs, beacon, or pseudonyms to a server which reconstructs and stores the pseudonyms in a format where user devices can query to ascertain their status or download them to their local devices for matching to confirm if the user is at risk or not. Any user whose device ID matches the published ones implied that he has encountered an infected person, hence, has a high chance of being infected. Figure 5 shows a pictorial representation of decentralized protocol.

Some of the protocols based on decentralized data management architecture include Apple-Google, DP-3T, PACT, and TCN. In real-life apps development, these protocols have been adopted by many countries around the world including Switzerland, Germany, and the United Kingdom.

**Apple-Google Protocol**: At the peak of the ravaging coronavirus pandemic, the two global technology giants—Apple and Google agreed to combine their efforts to solve the privacy-related challenges inherent in existing contact tracing protocols [35]. Being that Apple and Google are, respectively, the owners of the two major mobile phone operating systems, iOS and Android, their emergence in the scene of contact tracing brought about a major positive turnaround in the fight against COVID-19. The key feature of their protocol is the elimination of a central server that performs data storage, risk computation, and notification of risky users. Their protocol advocates that Bluetooth identifier beacon keys of devices that have had close contact with a user should be stored locally in an anonymous format. For any user that is diagnosed positive, the stored anonymous identifier beacon keys will be uploaded to a cloud server. To verify the status of every subscriber, there should be a periodic download from the cloud device which stores all the identifier beacon keys of users who tested positive of the disease. A key matching feature is integrated with the user app which compares the downloaded identifier beacon keys with those locally stored in the user device. A successful match of the keys implies that the user has come in contact with an infected patient, hence should self-isolate or seek medical advice.

**Figure 6:**
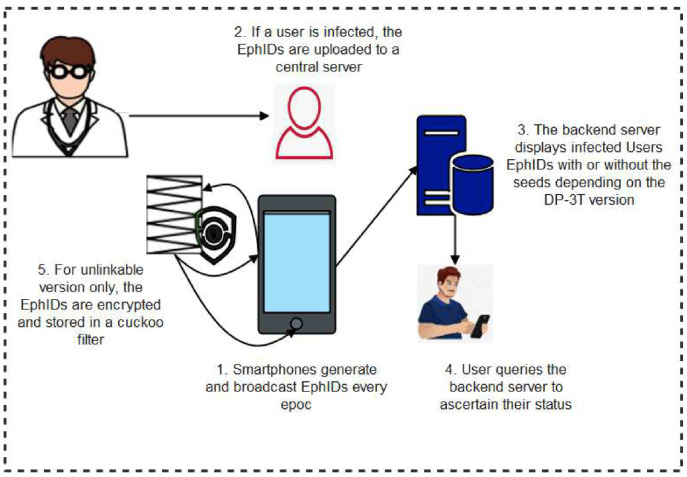
Conceptual diagram of DP-3T protocol.

**Distributed Privacy-Preserving Proximity Tracing (DP-3T):** DP-3T is powered by a group of international professionals, medical, technological, and legal experts whose common objective is to achieve user privacy preservation in contact tracing. It is more of a peer-to-peer contact tracing scheme that eliminated the overbearing control of central servers [36]. In DP-3T as shown in Figure 6, smartphones locally generate and broadcast ephemeral identifiers (EphID) for a given period called epoch which could vary between 10 min to 24 h before a new EphID is generated. Upon diagnosis of a patient, after due authorization by the relevant authority, the user uploads their EphIDs to a trusted backend server. To confirm the status of other users, the protocol is designed in a manner that every user periodically queries the central server to match the locally stored EphIDs with those stored in the backend server. A successful match shows that the user has come in contact with an infected user, hence could be at risk of the infection. There are three versions of the DP-3T protocol which include low-cost, un-linkable, and hybrid versions of this protocol. The low-cost version of the protocol computes and stores the EphIDs which are the hashes of the daily generated seeds [37]. This version disseminates the EphIDs along with the user seeds. The low-cost version is less bandwidth-demanding but is not fully free from user traceability attacks. The un-linkable version on the other hand is more bandwidth-demanding but has more user privacy features. Instead of disseminating the EphIDs along with the user daily seeds, the un-linkable version displays a hash of the seeds which is stored in a cuckoo filter. In this version, the user has the redact capability. This offers the user the flexibility to choose the period or time of the day which he wishes to display the EphIDs of their contacts. Finally, the hybrid design merged some features of both the low-cost and the un-linkable versions. In this version, the bandwidth requirement is lower than as it is in the un-linkable version yet, its privacy feature is higher than that of the low-cost version. In the hybrid version, a seed is generated for a defined window period. Upon infection of a user, only relevant seeds are uploaded.

**Privacy-Sensitive Protocol and Mechanism for Mobile Contact Tracing (PACT**): This is a decentralized contact tracing protocol proposed by researchers from the University of Washington [38]. The protocol which is closely related to DP-3T was designed in such a way that user devices generate private seeds through which random anonymous keys (pseudonyms) are computed and broadcasted as Bluetooth IDs. Each of the devices hears and records the pseudonyms of other nearby user devices. When a user tests positive for the disease, they upload their stored pseudonyms which represent the anonymous IDs of persons who came in close contact with them. A dedicated server reconstructs and publishes the pseudonyms in a comprehensible format for other users to infer their status by matching them with their respective pseudonyms. Any user whose device ID matches the published ones implies that he has encountered an infected person, hence has a high chance of being infected.

**CONTAIN**: This Privacy-Oriented Contact Tracing Protocol for Epidemics is similar to the DP-3T protocol in that a central server displays the details of infected users while other users confirm their status through the central server. In CONTAIN [39], user devices periodically beacon encrypted messages containing unique ID, timestamps, and random numbers that are stored locally. When a user is infected, their encrypted beacons are forwarded to a central server where they are displayed in an encrypted format. Other users get the data from the central server to ascertain their status. If users successfully decrypt the beacons, it implies they have been in contact with an infected person. Such a user is at risk, hence, should seek medical advice immediately. There is also another version of the protocol which is called a random beacon protocol. This is similar to the encrypted version only that the beacon is not encrypted but randomized. The beacons of an infected user are made public for other users to compare. If any of the beacons matches with the logs of any of the users, such a user must have been in close contact with the carrier, hence is at risk.

**Privacy-Preserving COVID-19 Contact Tracing App: A Zero-Knowledge Proof Approach (PPC):** In this protocol, subscribers generate both secrete and public keys and supply the public keys to the Government website during their daily registration. Health workers on the other hand acquire additional secret codes from their hospitals through which they generate group signatures on behalf of their respective hospitals. The contact encounters are recorded by user devices which acquire and store locally the hash of the Bluetooth IDs of nearby users’ Bluetooth active devices. Upon the confirmation that a user is infected, the patient sends the acquired anonymous data to a central health server where the health authority appends zero-knowledge signature before publishing the pseudo keys on the bulletin board for users to confirm their status. The users confirm their status by downloading the anonymous IDs from the bulletin boards to their devices which performs the decryption of the hashed data. A successful decrypting of any of the anonymous data implies that they have come in close contact with the infected person [40].

**Contact Tracing Application Using a Distributed Hash Table (CAUDHT):** The CAUDHT [41] protocol uses distributed hash tables (DHT) to the encrypted identities of users, hence sharing the data concerning the disease infections in a secure manner. In this protocol, an algorithm is created that encrypts the devices’ Bluetooth IDs using both secrete and public keys. While the secrete keys are kept with the user, the public keys are broadcasted as the Bluetooth IDs for other devices to store locally. Upon confirmation of infection, the user requests a blind signature from a central authority (server) before the contacts can have access the data in a distributed hash table.

**Temporary Contact Number (TCN) Protocol**: The Temporary Contact Number Protocol (TCN) is powered by experts from Stanford University and the University of Waterloo who came together to form the COVID-19 Watch team. It is a decentralized privacy-preserving contact tracing protocol whose source code is available online to the programming community. The protocol adopts Bluetooth low energy (BLE) as its proximity sensor. As users interact during their normal activities, their devices exchange their temporary contact number (TCN) which is a 128-bit number generated as a seed value of (a function) of the user BLE identity. The exchanged TCN is stored on the device’s local memory until a user gets infected and uploads them to the central server. Other users download and match the TCN locally on their devices. Only the device whose BLE seed value equals the TCN will have a successful matching, hence will be notified of the risk of the infection as a result of their close contact with an infected person [42].

#### 2.1.3. Hybrid Data Management Protocols

**Figure 7:**
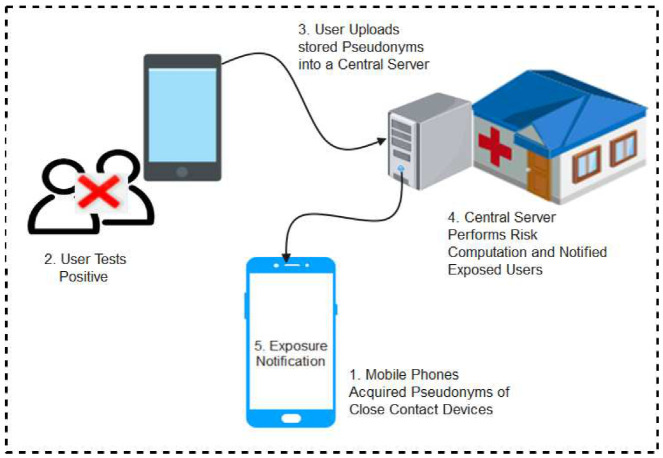
Conceptual diagram of hybrid contact tracing systems.

Additionally, the hybrid data management architecture balances the features of both centralized and decentralized protocols. In this approach, some functions are distributed while part is centralized. For instance, one device (such as a user’s mobile device or a stand-alone server) generates and manages the anonymous or pseudonyms keys/tokens while other systems perform risk analysis and notification of risky users as shown in Figure 7. This feature tries to close possible loopholes that may aid linkage attacks. Some of the protocols whose designs conform to the hybrid architecture include Contra Corona, Epione, and Desire protocols.

**Contra Corona:** This protocol is aimed at bridging the centralized–decentralized divide for Stronger Privacy [43]. It is a Bluetooth-based contact tracing mechanism against coronavirus where the server services are separated into matching and warning servers, respectively. Upon infection of a user, the anonymous contact details which were earlier stored in the user device are uploaded into the matching server. The matching server in turn performs risk computation using the uploaded data to identify the persons that came in close contact with the infected person. Once the data matching is completed, the list of risky contacts is forwarded to the warning server who sends messages notifying them of their risk level and the necessary actions to take. The key strength of this protocol is that it tries to block all clues that may contribute to learning who is infected or through who the infected was transmitted.

**Epione:** This is a lightweight Contact Tracing with Strong Privacy [44]. The protocol adopts the use of a pseudorandom generator (PRG) to generate random tokens in variance with its seed which are saved in the user’s mobile devices. The random tokens are exchanged when uses are in close contact. Users’ mobile devices also store all exchanged tokens from close contacts. When some users are diagnosed with the disease, encrypted PRG seeds (encrypted using the Epione server private key) from each of the infected users are handed to the health authority who thereafter sends it to the Epione server. The Epione server obtains the PRG seeds of the diagnosed patients through which it learns their tokens. For other users to confirm their status, their apps periodically query the Epione server which compares their tokens with those of infected persons uploaded by health the health provider. A match is an indication of having been in contact with an infected person.

**DESIRE:** In desire, the Third Way for a European Exposure [45], user mobile devices generate and locally save their private encounter tokens (PET) which are functions of their Bluetooth identifiers. If diagnosed positive to COVID-19, the user voluntarily uploads the stored PETs to a central server. The central server keeps a database of PETs of an infected person. Other users confirm their status through their apps which periodically queries the central server. The central server matches the respective mobile devices’ PETs with the stored PETs from infected persons. Any successful matching infers that the user has come in contact with an infected person.

#### 2.1.4. Mobile Applications (Apps) Based on Bluetooth Technology

Various mobile applications have been developed by countries around the world using the management architecture of Bluetooth low energy for contact tracing. Some of these apps include Singaporean TraceTogether, Australian CovidSafe, Canadian ABTracetogether, SwissCOVID, German-Warn-App, etc., here, we classified these mobile apps based on protocol architecture deployed for implementation. These include centralized and decentralized based mobile apps.

- **Centralized Architecture based Mobile Apps**

There are some existing national apps developed and deployed in the fight against COVID-19 which are deployed in line with centralized data management architecture. The Singaporean TraceTogether, Australian CovidSafe, Canadian ABTraceTogther, and Indian Aarogya Setu are good examples of the apps developed using the centralized architecture. These apps are briefly discussed below:

*The Singaporean Tracetogether*: The Singaporean Tracetogether is a very popular BLE-based contact tracing app that was among the foremost national interventions in the fight against Coronavirus. The Tracetogether app was developed by the Singaporean government technology agency (GoveTech), and the Singapore Ministry of health underpinning the centralized data management framework provided by the BlueTrace protocol [33]. *OpenTrace* which is the Tracetogether source code for both Android and iOS is available for the open-source community since March 2020. During encounters, users exchange 15 min TempIDs and store them locally in their mobile phone memories for 21 days. A positive tested user uploads the stored TempIDs to a central health authority server where the messages are decrypted and risky users contacted through their phone numbers. The Tracetogether was adjudged by the manufacturers as being fairly secure and privacy-preserving. This is because the app neither reveals user phone numbers and geolocation nor displays identifiable user details. These strengths initially attracted Singaporean citizens to Tracetogether to the extent that above 500 subscribers downloaded the app in one day.

However, within a short period, apathy against Tracetogether was observed among Singaporeans citizens. This occurred because there were some concerns raised against the application. First, it was alleged that the system compromised user privacy by keeping a record of peoples’ movement and interactions, hence user confidence in the app crashed. Secondly, it was alleged that the app drains phone batteries thereby making users uncomfortable [46].

*Australian CovidSafe*: CovidSafe is another contact tracing app that was developed leveraging on the centralized framework provided by BlueTrace protocol. It was powered by the Australian Health Authority and was released in April 2020 following the success stories of the Singaporean Tracetogether. The CovidSafe source code was released in May 2020. However, variations exist between the two protocols in the respective lifetimes of their TempIDs. While Tracetogether adopts the BlueTrace recommended 15 min lifetime of TempIDs, the CovidSafe resets its TempIDs every 120 min which could widen the replay attack windows [47]. Similar to the Tracetogether app, the CovidSafe app experienced an initial mass download of up to two million downloads within a day after release and above six million in a few weeks. Nevertheless, just like the Singaporean Tracetogether, some professionals still vigorously criticize the app. For instance, it is alleged that the privacy of CovidSafe users is not fully guaranteed. One major concern raised is that the company hosting the CovidSafe application may not be trusted. Many Australians are skeptical that Amazon Web Services (AWS), an American company may likely show allegiance to their country by illegally compromising their data if so requested by the United States of America. Furthermore, despite the number of downloads, the app was officially reported to have traced only about 200 contacts in the entire Australian nation [46].

*Canadian ABTracetogether*: The Canadian AB Tracetogether [48] launched in May 2020 was also developed leveraging on the centralized architecture provided by BlueTrace. The App is owned by Alberta Provincial Government and can be installed both on Android and iOS mobile phones. The subscription into ABTraceTogether is voluntary and users may quit at will. Similar to the Tracetogether of Singapore and the CovidSafe of Australia, the AB Tracetogether app of Canada was fully embraced by the people of Alberta. Within one week of its deployment, the app recorded 140,000 downloads.

In this protocol, the central server assigns a permanent ID to every device upon registration. The IDs are encrypted and broadcasted as Bluetooth IDs. During interactions, each system logs the encrypted version of the Bluetooth IDs sensed from other users’ mobile phones within a 2 m distance for up to 15 min. When a user is diagnosed with COVID-19, the stored data is uploaded to a central server through which their close contacts are identified and informed of their risk level. The central server achieves this by decrypting the uploaded Bluetooth IDs to decipher the permanent ID through which the user is identified and contacted. The AB Tracetogether app has been adjudged by experts to be safe for contact tracing against infectious diseases.

However, the major weakness of the app is that it is prone to a single point of failure vulnerability just like every other app designed based on a centralized architecture. Furthermore, the AB Tracetogether app does not work well in Apple iOS, at least in the current version [49].

*Indian Aarogya Setu*: The Indian Aarogya Setu [50], is a contact tracing mobile app developed by the Government of India in the fight against COVID-19. The application was among the most popular contact tracing apps in the world with over one hundred million downloads in about forty days of its launch. It combines Bluetooth and GPS technologies in mobile phones to perform its contact tracing. To subscribe, users must provide an Indian mobile number and other relevant details. The system records Bluetooth IDs and location details of every encounter and forwards the same to a central server in an encrypted format. When a user is diagnosed, the system notifies all their contacts of their risk level. Its use is mandatory especially for professionals working in both public and private establishments in India.

The Government of India has promoted the Aarogya Setu app explaining that it exhibits a reasonable level of user privacy, security, and transparency. They further involved Indian professionals for security audits and enhancement to improve acceptability and user trust. This led to a well-articulated privacy policy document that is available for public scrutiny. Consequently, there is an enhanced adoption rate of up to 150 million users making Aarogya Setu one of the most downloaded contact tracing apps [51].

However, similar to other apps developed in line with centralized network architecture, the Aarogya Setu app is prone to single point of failure vulnerability just like every other app designed based on a centralized architecture. Furthermore, since the app is built around user phone numbers, there is a likelihood of attacks through user phone numbers [52].

- **Decentralized Architecture based Mobile Apps**

We discuss some of the apps developed following the decentralized architecture. Some of such apps include the SwissCOVID, the German Corona-Warn-App, and the NHS COVID-19 App of the United Kingdom.

*SwissCOVID*: The SwissCOVID app [53] is a legally approved decentralized contact tracing app developed following the Apple–Google and the DP-3T frameworks. The app which was released on the 25 June 2020 is powered by the Swiss Federal Office of Public Health (FOPH) in collaboration with some organizations such as the Federal Office for Information Technology, Systems and Telecommunication (FOITT), Federal Institutes of Technology in Zurich (ETH), Lausanne (EPFL) and the Swiss company *Ubique*. It uses Bluetooth technology for proximity detection in which Bluetooth data of people who have come in contact with a user are stored locally in their mobile devices. In this scheme, there is no central server where data is uploaded for risk-level computation and notification of risky users. Rather, both proximity detection, data storage, and notification of risky individuals are performed by the user devices. Upon positive diagnosis of a user, a *Covidcode* (also known as release code) is issued to the user by the health authority empowering him to activate the notification feature of the app thereby enabling the user to send warning messages to those who have come in contact with them. However, a critical review of the app that was carried out by [54] reveals that this app is vulnerable to false positive and linkage attacks.

*German Corona-Warn-App*: Launched on 16 June 2020, the German Corona-warn-App [55] is another decentralized contact tracing app developed leveraging the Apple–Google framework. Its development is powered by the federal ministry of health in collaboration with some technical institutions such as Deutsche Telekom and SAP. The system generates varying (yet remembered) Bluetooth IDs which are broadcasted for handshakes with nearby Bluetooth active mobile phones. Each user device stores the Bluetooth ID of any mobile device that comes in contact with it. When one of the users is diagnosed with the disease, the stored IDs are voluntarily uploaded to the central system. The central system only acts as the storage of IDs of infected people and each user periodically downloads the stored IDs to enable their mobile devices to compare to find out if there is a match. If there is an ID match, it implies that the user has encountered an infected person. In such a situation, the user app computes the risk level using the encounter distance, duration of the encounter, and other relevant indices before issuing a notification. However, analysis of this app shows that the app is vulnerable to revealing user identity and possible false-positive attacks [56].

*NHS COVID-19 App of United Kingdom*: The National Health Service (NHS) of the United Kingdom (UK) has powered the development of a decentralized Bluetooth-based contact tracing application called NHS COVID-19 App [57] which was launched on the 24 September 2020. It was developed following the Apple–Google exposure notification and logging framework using Bluetooth technology. The app was deployed for residents of England and Wales of ages 16 years and above where over 21 million downloads were recorded.

If a user tests positive for the coronavirus, the app notifies their close contacts to self-isolate and can help the user to request a test. A special feature of the app is the integration of a QR code that notifies a user of any visit to a high-risk location. However, a study has shown that the app is vulnerable to user privacy leakage [58]. We also summarized the features of Bluetooth-based contact tracing apps and Protocols in Table 2 and Table 3, respectively.

### 2.2. Global Positioning Systems (GPS) in Contact Tracing

Global positioning system (GPS) is a satellite-based positioning technology that can provide real-time object localization anywhere on the surface of the earth. It is made up of three major segments: orbital satellites, the control stations, and the user devices (GPS receivers). The orbital systems are comprised of over 24 satellites fitted with stable clocks in space for time synchronization. They are controlled through one major control station located at the Colorado Springs Air force base, Colorado, United States of America. Other unmanned control stations are spread across different locations of the world including Hawaii, USA, Ascension Island in the Atlantic Ocean, Diego Garcia in the Indian Ocean, and Kwajalein in the Pacific Ocean [3].

**Figure 8:**
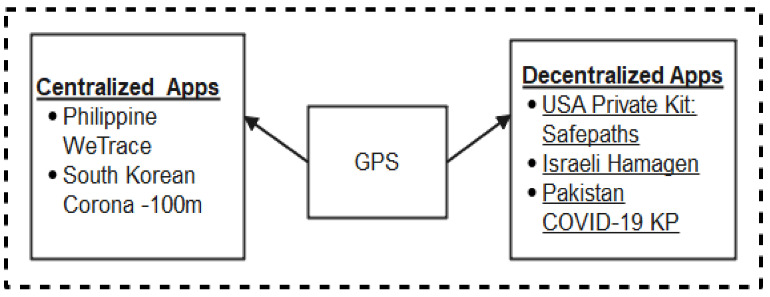
Global Positioning System-based Apps.

Today, GPS systems are being exploited in many non-military operations such as wireless video processing and monitoring, navigations, surveying, internet services, and location tracking [59]. The GPS can track more than one object and determine their respective distances relative to each other, also taking record of their periods of interactions. This special feature has positioned GPS as a choice technology for contact tracing systems. Moreover, researches have shown that there are many weaknesses inherent in GPS-based systems. First, GPS systems reveal device identity and locations, therefore, are prone to security and privacy-related vulnerabilities. Furthermore, it has been observed that GPS-based systems suffer from poor co-location accuracy, high battery consumption and are not suitable for indoor applications [60,61,62]. Despite these weaknesses of GPS-based systems, there are some Apps developed leveraging GPS technology in the fight against the coronavirus. We briefly discuss these apps classifying them into two: centralized and decentralized apps as shown in Figure 8.

#### 2.2.1. Centralized Architecture-Based GPS Mobile Apps

*Philippine WeTrace*: WeTrace [63] was developed by a team of experts in Genni Hut Technologies Incorporated of the Philippines. Later, it was adopted and made compulsory by the Cebu province authority for use by its residents. It is a GPS-based system designed to detect people within the Cebu Province who has some COVID-19 related symptoms such as catarrh, cough, difficulty in breathing, and fever. The system uniquely identifies users with a QR code or device ID number. It reports its findings to relevant health authorities, performs mapping of infected persons, and keeps logs of their movements and locations. However, users have criticized the app as being poorly developed, delays in loading, and draining phone batteries.

*South Korean Corona-100 m*: The South Korean Corona-100 m (Co100 app) is a privately developed app that utilizes data from the Government database to notify subscribers of diagnosed patients’ whereabouts. The system acts as a digital perimeter fence of about a 100-m radius and alerts users if any diagnosed patient encroaches the borderline or is within the 100-m radius. Furthermore, the Corona Map utilizes Government data to keep track of diagnosed patients’ movements or locations visited. The major weakness of these apps is that there is a possibility of user privacy abuse since the app makes public patient’s diagnosis date, nationality, age, gender, and prior locations [64].

#### 2.2.2. Decentralized Architecture-Based Mobile Apps

*Israeli Hamagen*: The Hamagen is a contact tracing app fully endorsed by the Israeli ministry of health as a veritable tool to combat COVID-19. It is a GPS-based solution that compares users’ GPS logs with data sent from the ministry of health which represents the locations visited by infected persons. Where there is a likelihood that the user has come in contact with an infected person, the app notifies the user stating the exact location and time. Where the user is convinced that such occurred, the notification is accepted and other steps are taken for further diagnosis. On the contrary, the notification is rejected and normal life continues [14]. However, this app most likely suffers poor accuracy and will not be suitable for indoor applications as applicable to GPS-based systems [60,61].

*Iranian AC19*: The Iranian AC-19 is a contact tracing app that employs GPS to determine the user’s location. Launched in March 2020 by the Iranian ministry of health in the fight against COVID-19, the app provides a platform for self-diagnosis by citizens, a feature that is aimed at reducing congestion in the country’s health facilities [65].

However, there are concerns that the app collects and uploads citizens’ sensitive data such as phone numbers, names, addresses, dates of birth, and movement records to the central server. Consequently, the Government has been criticized by some experts as being unduly utilizing such data to track users’ movement and invade citizens’ privacy, hence, the removal of the app from the Google play store [66].

*The USA Private Kit-Safepaths*: Safepaths [67] combines trails from Bluetooth and GPS to provide a platform through which users can determine if they have come in contact with a person infected with the coronavirus. It is an open-source application powered by the Massachusetts Institute of Technology (MIT) which is aimed at providing a free and privacy-preserved contact tracing solution against COVID-19. The app collects subscribers’ location information by keeping an encrypted copy of 5 min interval logs within the last 28 days discarding older data. In the early version, Users are expected to upload their locations to the health officials if diagnosed positive but the later version has a feature to notify their close contact of their risk. It comprises both mobile phone applications called privateKit and a web application called safe places.

*Pakistan COVID-19 PK*: The Pakistan COVID-19 PK was developed by the Ministry of Information Technology and Telecommunication in collaboration with the National Information Technology Board of Pakistan. It is an application that is fitted with a dashboard that keeps citizens informed of the total infected persons arranged in the province by the province before providing a summed figure for the entire country. It also has self-assessment and some notification features such as radius alerts and personal hygiene reminders. The system also has some interactive features such as Chabot and sensitization videos. However, the developers did not provide the privacy details of the app [68]. Features of the centralized and decentralized GPS-based contact tracing systems are also summarized in Table 4 and Table 5, respectively.

### 2.3. Artificial Intelligence (AI)

Artificial intelligence (AI) is the creation and training of devices—robots and other smart machines to become intelligent enough to be able to perform human-related activities such as learning, reasoning, and self-correction [20]. There are many application areas of artificial intelligence ranging from speech recognition, semantic information processing, language translation, learning and adaptive systems, pattern recognition, modeling, robotics and games, healthcare, automotive, economics and computer networks, etc. [69].

More so, AI has been applied widely in the fight against infectious diseases such as COVID-19. Areas of its possible application include medical diagnosis [70], virus transmission modeling, and forecasting (Hu et al., 2020; Jiang, Coffee, Bari, Wang, and Jiang, 2020; R. K. Singh, Rani, Bhagavathula, and Sah, 2020), biological data analysis for drug discovery [71], etc. For instance, in Canada, the *Blue dot* was used to predict the outbreak of the coronavirus before it arrived [72]. Data from social media were also analyzed to provide intelligence concerning the COVID-19 outbreak before the World Health Organization (WHO) announced the outbreak [73]. Furthermore, deep learning modeling was employed to assist in the detection of COVID-19 disease on X-rays films [74]. The model when demonstrated with 260 images showed very high accuracy. This result suggests that the model could assist health workers in the early diagnosis of COVID-19 cases.

Furthermore, technology experts and researchers have continued to point out the high potential of artificial intelligence in contact tracing against infectious diseases [11,12,69]. For instance, Facedrive Inc., a Canadian organization who in partnership with some researchers from the University of Waterloo has announced its plan to combine Bluetooth and artificial intelligence technologies in the development of TraceScan, a contact tracing and risk alerting system [75]. Similarly, Volan Technology, a company that provides hotel security and similar services has launched an artificial intelligence-based contact tracing, social distancing, and temperature monitoring system. Their system is a modification of the technology originally designed and piloted for emergencies in schools, hotels, and other workplaces but the advent of COVID-19 has opened the door for its application in contact tracing [76]. Despite the much-talked-about potentials of artificial intelligence in contact tracing, only a few fully tested and nationally recognized artificial intelligence-based contact tracing apps have been deployed. Some of those apps include The Chinese Alipay and WeChat mobile app and the StayHomeSafe of Hong Kong. Their features are AI-based contact tracing systems are summarized in Table 6.

*ChineseAlipay and WeChat Mobile App*: The Chinese Alipay and WeChat mobile applications are quick response-based systems that rely on self-inputted data by users. The user scans a government-owned QR scanner over their mobile device before being granted access to public places. The system compares the QR acquired information with health authority records domiciled in a central server before assigning the users one out of the three color codes of green, yellow, or red. The color codes define the infection risks of users ranging from free from the infection (green), status not yet known but at high risk (yellow), and confirmed carrier (red). The infection status determines the level of freedom of movement of such individuals. The government requires citizens to strictly obey the set down rules or face serious sanctions [66]. These functions are performed by allowing users access to only adjudged safe places and also keeping a log in the central server for notification of users in case of a positive diagnosis of their close contacts. However, there have been privacy-related concerns in using this system since the identities of the users are required at the point of enrollment [77].

*LeaveHomeSafe of Hong Kong*: The LeaveHomeSafe of Hong Kong is a QR code-powered contact tracing application that was launched on 16 November 2020. The download of the app is voluntary except for overseas returnees who are mandated to wear a smart wristband integrated with the LeaveHomeSafe app within the first fourteen days of their arrival. The app keeps the log of public places and taxis visited or boarded by the users taking special note of the date and time [78]. It implied that once a user visits a public place or boards a taxi, he/she clocks in of public places or taxis by scanning their QR code and clicks the leave button when leaving the venue. If a user is diagnosed with COVID-19, the app notifies users who visited the same place with the infected person at the same time. The user can also upload their record to the central server for further use by the health authority. There are, however, some privacy concerns against the app. For instance, despite the assurances of the Government, the citizens are not certain of the safety of the data collected and are afraid of being tracked using the app.

However, studies have shown that there are limitations of artificial intelligence-based contact tracing techniques. For instance, as was noted in [25,79], artificial intelligence is only effective if the relevant data needed for the analysis or the training of the intelligent agents is available. Unfortunately, at the early stage of disease outbreaks, such data are not usually available [80].

### 2.4. Other Technologies for Contact Tracing Systems

In this subsection, we review other technologies recently proposed by researchers for the implementation of contact tracing systems and also attempt to highlight the features that make them suitable for such applications. Some technologies include Wi-Fi, Smartphone Magnetometer Traces, Near Field Communication (NFC), Radio Frequency Identifier (RFID), and Blockchain technology.

#### 2.4.1. Wireless Fidelity (Wi-Fi) in Contact Tracing

Wireless fidelity (Wi-Fi) is an IEEE 802.11 standard-based wireless communication technology that connects devices such as mobile phones, tablets, and computer systems to other network devices or the internet at a high speed without the use of network cables [81,82]. Wi-Fi signals are transmitted at frequency ranges of 900 MHz, 2.4 GHz, 3.6 GHz, 4.9 GHz, 5 GHz, 5.9 GHz, and 60 GHz bands at the speed of up to 150 Mbps. The more recent upgrade in wireless fidelity is version 6 (Wi-Fi 6) which is supported by IEEE 802.11ax standards [83].

**Figure 9:**
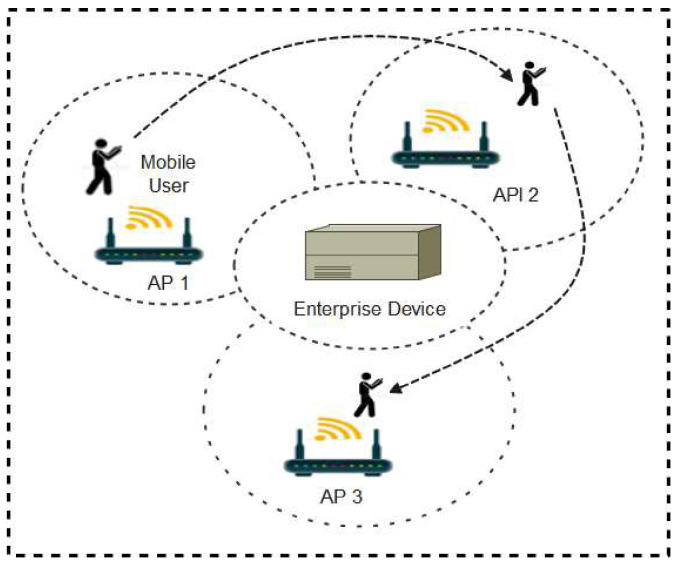
Wi-Fi setup in a large network.

In a Wi-Fi environment, wireless access points (AP) automatically advertise their service set identifier (SSID) through radio signals broadcasts for other Wi-Fi enabled devices within the area of coverage to connect for onward communications [84]. In a large Wi-Fi setting such as a University campus, multiple access points are strategically installed to ensure maximum coverage. A key feature of Wi-Fi technology is that as users move from one location to another, the user devices keep reconnecting to nearby access points thereby creating Wi-Fi fingerprints or digital traces in the enterprise device as shown in Figure 9.

The continuous handshakes between APs and user devices as the user moves from one location to the other is an important index for contact tracing. There are two major approaches to developing Wi-Fi-based contact tracing systems. The two methods are the client-centric and the network-centric approaches. The network-centric approach entails direct analysis of the user logs stored in the enterprise devices while the client-centric method requires the development of a mobile app that will acquire this information and perform the analysis in the user’s mobile devices [85]. Various contact tracing protocols have been developed using the Wi-Fi technology. These protocols are explained below. 

*WifiTrace*: A foremost Wi-Fi-based study was presented by [86] where the authors proposed a network-centric contact tracing Protocol captioned WiFiTrace which employs a graph-based analytic tool for contact tracing. Infected users’ network logs are used to plot a trajectory graph showing their movement history relative to the people that came in contact with them and for how long they interacted. Although WiFiTrace protocol could aid contact tracing, the authors admitted that it could only supplement the traditional method but cannot be fully depended upon for effective contact tracing during an infectious disease outbreak.

*Encounter-Based Architecture for Contact Tracing (ENACT)*: On the other hand, Prasad and Kotz [87] proposed a client-centric contact tracing protocol named Encounter-based Architecture for Contact Tracing (ENACT). A mobile contact tracing application is developed which acquires the user’s event tags containing the MAC address of access points connected and user location. The AP gives a footprint of locations visited by the users. Upon diagnosis of a user, the ENACT server performs a matching of the tags which reveals people who had close contacts. A similar study was carried out by [88] where the authors proposed VContact, a Wi-Fi-based contact tracing protocol. In this protocol, Wi-Fi provides the communication platform for some internet of things (IoT) devices such as smartphones, smart wristwatches e.t.c which perform the sensing of close devices. Data acquired is uploaded to a central server for further analysis. The unique feature of this work is that it puts into consideration the virus lifespan.

However, there are some weaknesses inherent in Wi-Fi-based systems. The system can only perform within the area of coverage of the Wi-Fi network. This made Wi-Fi a choice technology for projects in the confined environment but not for national deployment. Furthermore, where users own more than one device or fail to connect their devices to the Wi-Fi network deliberately or unknowingly, their movement cannot be monitored or traced.

#### 2.4.2. Smartphone Magnetometer Traces

Smartphones have been observed to maintain high linear correlation in their magnetometer traces if positioned at close range [89]. This discovery is being harnessed in human proximity detection against infectious diseases. Being that magnetometer traces neither reveal devices’ identities nor locations nor does it require additional infrastructure, this method possesses high potentials for conforming to user privacy-preserving designs. The experiments reported in [60] show that the magnetometer traces coefficient is strong at distances of about 1–2 m between the smartphones both for static and dynamic coexistence. A few studies have been carried out to evaluate the suitability of magnetometer traces in contact tracing against infectious diseases. The study presented in [90] which attempted to evaluate the usefulness of magnetometer traces in proximity detection is a good example. In the study, magnetometer sensing apps were developed and installed in some android smartphones including Samsung Galaxy S5, S6, S8, and LG G3 and G4, and synchronized with Network Time Protocol (NTP). Three locations each in five different buildings on campus were selected for the experiments and magnetometer traces were collected six times in each of the locations. The magnetometer sensing apps submitted their stored traces to a centralized server for analysis and computation. The result of the analysis shows that the smartphone magnetometer-based method is accurate and could be adopted as a clinical tool for contact tracing. Similarly, Kuk et al. [61] reported a study to determine the relationship between clarity of magnetometer traces, sampling rate, and smartphone battery consumption. The study utilized magnetometer traces independently generated by different people across different countries for their analysis. The result shows that although existing magnetometer-based systems employ high frequencies of about 10–200 Hz, apps designed using the frequency of 1 Hz is more energy-efficient, yet it is sufficient to detect the correlation of smartphone magnetometer traces. This implies that developing apps at a sampling rate of 1 Hz is sufficient for proximity detection and will prolong smartphone battery usage time.

While we note some of the challenges inherent in magnetometer-based methods including that magnetometer traces are susceptible to distortions within ferromagnetic [60] and that battery consumption is still a challenging issue in magnetic traces-based proximity detection systems [61], we opine that the potentials in this technology are yet to be fully harnessed especially in the area of contact tracing against infectious diseases.

#### 2.4.3. Cellular Networks (CN) in Contact Tracing

**Figure 10:**
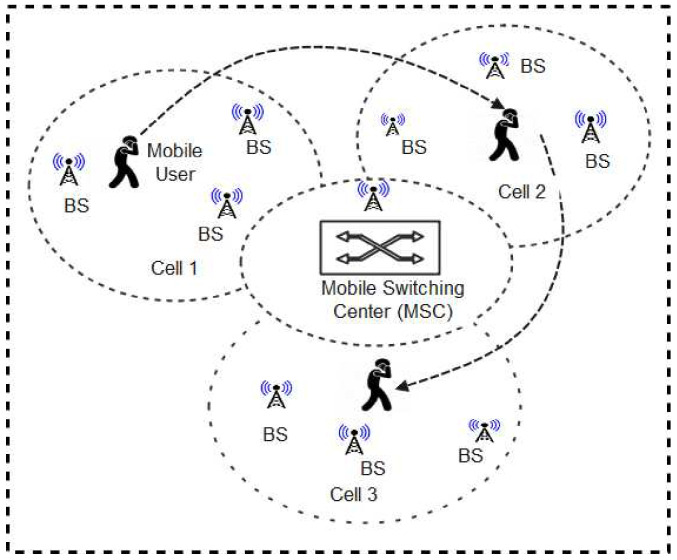
A typical cellular Network architecture.

Cellular Networks (now referred to as mobile phone networks) are modern technologies for mobile communication. Generally, the architecture of cellular networks is as shown in Figure 10. Nordic mobile telephone (NMT) was the pioneer cellular system ever developed and launched in some countries including Denmark, Finland, Norway, and Sweden [91]. This was followed by the development of other wireless mobile networks such as first-generation (1G), second generation (2G), enhanced second generation (2.5G), third generation (3G), and fourth generation (4G) [92,93,94]. Lately, the fifth generation (5G) network was developed while other newer generation networks are also being proposed [95]. In these emerging networks (5G and beyond), enabling technologies such as Multiple-Input Multiple-Output (MIMO) [96] are utilized to enhance the multiplexing techniques, frequency spectrum bands, network throughput, and spectral efficiency relative to older generations of cellular networks. This implies that they are expected to provide a more robust, flexible, and efficient wireless communication platform capable of accommodating the high volume of data generated from the emerging IoT-driven wireless technology sector.

Fascinatingly, cellular technologies particularly the 5G network has been identified to be beneficial in the fight against COVID-19 [97,98,99]. Furthermore, 5G has been proposed for contact tracing. For instance, Zhang et al. [100] proposed PTBM, a 5G-based privacy-preserving contact tracing protocol linked with BlockChain-based medical applications. In this system, user devices are installed with the contact tracing apps and connected with 5G networks which enable users to perform contact tracing without infringing on the privacy bounds of other users. Similarly, Rahman and Khan [101] proposed a contact tracing framework using user position data provided by the cellular network. The protocol is utilized to pinpoint high-risk areas of COVID-19 and trace the contact of infected persons. The phone numbers of all exposed users are securely stored in a central server managed by the health authority. Those exposed users are thereafter notified of their risk and advised to seek medical attention. Contact tracing is therefore performed by retrieving the phone numbers of people whose mobile phone data reveals that they have come in close contact with an infected person. A related study was carried out by [102] where the authors developed a new framework that utilizes logs of the 5.85 million cellphone users in Shenzhen city for determining the intra-urban risk of the dengue fever disease. A human trajectory map was developed which gave insight on a better intervention strategy against the disease. This suggests that the data was helpful in the fight against dengue disease. Similarly, Farrahi et al. [103] proposed a contact tracing scheme using communication traces obtained from mobile phones providers. The study lasted for over a nine month period using a dataset of 72 students whose physical interactions as well as their mobile phone communication traces were known. The result of the work suggests that this approach could aid contact tracing during an outbreak of epidemic diseases. However, the accuracy of cellular network-based systems is a major challenge for its application in contact tracing and social distancing systems. High precision measurement of a few meters (2 m for example) may be difficult to achieve [90].

#### 2.4.4. Radio Frequency Identifier (RFID) in Contact Tracing

RFIDs are real-time location systems that use unique codes to perform automatic and contactless objects identification even if not aligned in a line of sight. The three major components of RFID devices include RF tags, antennas, and readers.

The RF tags also known as transponders are chips programmed with unique codes and fixed on objects or devices for identification. The RF readers have inbuilt memory devices for the storage of unique identity codes. The communication between the RF tags and readers is made possible by the antennas fitted in both devices.

The tags respond to queries from RFID readers by supplying their unique codes and other accompanying data to the reader. There are two major classes of RFID based on the type of transponder-active and passive RFID. An active RFID is characterized by in-build batteries for its operations. On the other hand, the passive RFID systems are dependent on energy sources from the RFID readers for them to be powered. To obtain object identity, the RFID readers also called interrogators query the RFID tags who in turn supply its identity details. The interrogators could be RFID read-only or read-write readers. The read-only readers can only obtain programmed identity codes from the RFID tags while the read-write readers can be used to also program a blank tag or edit existing identity code [104]. There are many application areas of RFID systems especially in object localization and tracking, industrial application, supply chain, retailing, financial exchanges, and access control. RFID devices possess some attractive features that made them appealing to technology experts and researchers. First, RFIDs obtain the identity of objects in an automatic and contactless manner. Furthermore, it does not depend on line of sight for its operations.

These features perhaps have attracted researchers to explore their suitability in contact tracing against infectious diseases. Some studies have been carried out to compare the effectiveness of the RFID-based contact tracing approach with the conventional electronic medical record (EMR) method. For instance, Hellmich et al. [105] and Nibras et al. [106] evaluated the effectiveness of real-time location systems (using RFID) vis-à-vis the traditional Electronic Medical Record EMR methods for contact tracing against pertussis disease were carried out. The study shows that the RFID-based method produced double of the EMR result which indicates that RFID has a very high potential in contact tracing against infectious diseases. During the outbreak of COVID-19, a similar study reported in [107] validated the earlier findings in [105]. There are so many other RFID-based studies in contact tracing. For instance, the study reported in [108] proposes a combination of RFID and GPS technologies to achieve effective contact tracing solutions against infectious diseases. Furthermore, Bian et al. [109] integrated RFID with Blockchain for contact tracing.

However, it should be noted that even though RFID technology has the advantages of the low cost of deployment and not being limited by line of sight, some inherent weaknesses need to be considered and enhanced for its effective application in contact tracing is achieved. One of such weaknesses is that RFID tags are limited in storage, hence may not accommodate many security codes. This implies that the security of RFID systems may not be fully guaranteed. In addition, RFID tags are limited in battery, hence may not be powered over a long time without incurring the cost of battery replacement [110].

#### 2.4.5. Near Field Communication (NFC)

The Near Field Communication System (NFC), which was founded by a combined effort of Sony and Philips, is a technology developed leveraging the RFID technology. NFC, therefore, shares a similar interface and protocol with RFID making both technologies compactible. It is a wireless communication protocol for objects at a close range of fewer than 4 cm at a transmission speed of about 424 kbps. It can communicate between an active and passive device or between two active devices. The communication between the NFC devices is achieved using the magnetic coupling technique [111].

NFC is applied in smart technologies such as access control systems and wireless payment and ticketing systems. Therefore, it can be said that NFC is a technology that provides a seamless, fast, and reliable platform for device communication and data exchange [111]. NFC is among the emerging technologies with the potentials for deployment in contact tracing systems. However, its applicability is yet to be investigated [15].

#### 2.4.6. Internet of Things

The term internet of things (IoT) was coined by Kevin Ashton in 1999 when he envisaged a world where physical objects will have internet capability to support human-to-machine and machine-to-machine communication [112]. This concept entails that objects are fitted with intelligent devices and communication capabilities to achieve remote data transfer and/or control. Interestingly, the IoT industry has grown rapidly as there is massive integration of sensors and actuators to the network thereby exponentially multiplying both the number of subscribers and also data generated. IoT application spans many fields of human endeavors. As was outlined in Nord et al. [113], there are various application areas of IoT to include but are not limited to energy, transportation, logistics, industry, supply chain, agriculture, health, and smart environment (homes, city, office, car, etc.). However, recent studies show that the emergence of COVID-19 has shifted investments on IoTs to the health and related sectors believed to be relevant in the fight against the pandemic [114].

Consequently, experts have proposed various frameworks for the effective application of IoTs to combat COVID-19. For instance, Roy et al. [115] proposed a novel IoT-based protocol that can detect basic symptoms of the COVID-19 disease and also perform efficient tracking of the disease spread by identifying infection clusters. This framework helps both in fighting the disease and in the equitable distribution of scarce materials such as protective equipment during the pandemic period. Similarly, an IoT-based framework has been employed for real-time monitoring of users against known symptoms of COVID-19. The framework also performs a follow-up monitoring of patients who have recovered from the disease. In addition, the framework collects and analyzes relevant data to further understand and reveal the characteristics of the virus which could be useful in its diagnosis and treatment [115].

However, despite the advances made so far in the field of IoT, experts believe that its potentials are still under-explored [116]. Therefore, the application of IoT to achieve privacy-preserving contact tracing against infectious diseases remains an open research direction.

#### 2.4.7. Blockchain Technology

Blockchain (BC) which was first introduced by Satoshi Takemoto in 2008 is a decentralized database originally designed for financial-related applications. It is the underlying technology of bitcoin—a peer-to-peer electronic cash system in which a virtual currency called bitcoin is circulated in the online economy without a central controller [117]. This approach entails the mutual performance of transactions using a distributed online ledger. If the transaction meets the requirement, it is unanimously validated by the nodes (called miners) in the network [118]. The transactions once validated are linked (or chained) to older transactions forming shapes that look like a group of blocks chained together, hence the name Blockchain. Hash as was explained by [119] is a mathematical algorithm that produces a string of characters called hash value which is used to sign digital signatures as a means of validating that the requestor is the rightful person. Blockchain can be classified as public (permission-less), Private (permissioned), or Consortium (hybrid) Blockchain technologies [120]. The public Blockchain (PBC) is open for anybody to join. PBC is fully decentralized and transactions are open for all to read and write to. Prove of work (POW) is the consensus mechanism in which every node will participate making the process resource-demanding and time-consuming. An example of public Blockchain is bitcoin. Consortium Blockchain is partially decentralized while private Blockchain is fully centralized. Private Blockchain and consortium are restricted to participating organizations. Consensus is performed using proof of stake (POS) or other, variants of consensus mechanism in private and consortium Blockchain.

The distributed data storage and management feature of Blockchain is appealing and is expected to play a major role in resolving the privacy issues inherent in data management architectures in the existing contact tracing applications. The study reported in [109] lays credence to this assertion. In this research, the authors evaluated the feasibility of integrating Blockchain with IoT devices (RFID) in the deployment of contact tracing systems. Their prototype was developed using Ethereum Blockchain taking advantage of its smart contract. The evaluation performed on the prototype shows that the approach is cost-effective. A similar framework was proposed in [109] using a public Blockchain network (PBN) where an infected person can share his contact list by initiating a transaction. Upon successful approval and addition of the new block, other users can confirm their status by initiating a query transaction on the Blockchain network. Another Blockchain-based contact tracing framework was proposed by [121] where the authors opined that with their framework, infection risk for international travels is reduced. Their proposal also ensures that contact tracing of users can be achieved in a privacy-preserving manner.

#### 2.4.8. Software-Defined Networking

Software-defined networking (SDN) is an emerging paradigm in the networking ecosystem that employs standardized network application programming interfaces (API) for network configuration, data storage, and data sharing. It separates the network control from the data forwarding functions thereby creating a platform for independent programming of the network control This implies that SDN is a three-tier architecture where applications and high-level network instructions occupy the top tier, the controller in the middle tier while the bottom tier houses the infrastructure layer where both the physical and virtual switches are located. One major feature of SDN is that it is an open standard architecture that eliminates vendor-specific dominance and control [122].

Interestingly, due to the open standard feature of SDN, experts consider it as a potential technology for multi-domain applications such as vehicular ad-hoc networks (VANETs) [123], big data applications [124], mobile ad-hoc networks [125], and the internet of things [126,127]. Furthermore, SDN has been proposed as a veritable technology that can provide a high quality of service in providing telemedicine services during the COVID-19 pandemic [128].

In addition, the emergence of COVID-19 introduced a shift to a ‘new normal’ especially on how resources are accessed over the internet. For example, people work from home, shopping has to be performed online and lectures are attended remotely. This paradigm shift comes with some constraints such as multi-domain interoperability, scalability, and security of network systems. Unfortunately, the existing network infrastructures are yet to meet these requirements. To solve this problem, experts are proposing that SDN technology may provide the needed dynamism for such complex scenarios [129]. For example, Jung et al. [130] proposed an SDN-based platform for monitoring infected persons who have their smartphones installed with the virtual IoT app. In this framework, the controller serves as the central point where location information and other relevant data from the respective virtual IoT nodes are collected.

Moreover, as has been observed earlier, contact tracing systems are most susceptible to architecture-related issues such as single point of failure, data security, and user privacy issues. Consequently, a lot of research efforts have been expended to enhance the architecture of contact tracing systems. Therefore, in our opinion, since SDN is an emerging network architecture, its potential in this regard should be explored by the research community.

For clarity purposes, we summarized the features of contact tracing protocols based on these technologies in Table 7.

### 2.5. Proximity Estimation Techniques in Contact Tracing Systems

The effectiveness of contact tracing schemes depends on the accuracy of the proximity detection methods. For instance, the accuracy of IoT-based systems such as [131,132] are dependent on the precision of the imbedded sensors. It follows that an efficient proximity detection technique will produce a highly accurate system devoid of the common errors in contact tracing systems including false positive, false negative, and other errors [133]. Proximity estimation is a key step in proximity detection because it tries to compute the distance between one object and the other in space. It therefore implies that proximity detection algorithms rely on the precision of the proximity estimation technique for its effectiveness.

Some conventional proximity estimation techniques include the time of arrival (TOA) [134], time difference of arrival (TDOA) [135] angle of arrival (AoA) [133] have been implemented in various contact tracing systems. However, notwithstanding that these techniques offer relatively high accuracy in proximity estimation, their setup is complex requiring multiple antennas and high precision synchronization [135]. Consequently, other proximity estimation methods such as the RSSI, GPS, and computer vision are being employed in contact tracing systems.

#### 2.5.1. RSSI-Based Proximity Estimation Technique in Contact Tracing Systems

The received signal strength indicator (RSSI) technique estimates object positions by measuring the difference in signal power between the source and the destination. This technique employs a path-loss model [136] where the range can be expressed as:

RSS = A − 10nlogd(1)

where A is the received signal power 1 m away from the transmitter, d is the distance from the transmitter to the reference point and n is the path-loss exponent of the environment.

There are two main approaches to the RSSI proximity estimation namely, RSSI trilateration and the fingerprinting methods [137,138]. The major weakness of the RSSI methods is that the performance is largely dependent on factors that vary once there is an environmental change. For instance, if a new infrastructure such as furniture, refrigerator, etc. are introduced to the same environment, or the distance estimation is performed in an entirely new location, the RSSI value changes due to the shadowing, shading effects, and the multipath losses in the different environments [137]. Moreover, despite the errors in RSSI-based techniques, it is still a very popular proximity estimation method in contact tracing systems [139]. Consequently, experts have proposed various schemes for enhancing the accuracy of RSSI-based proximity estimation techniques. Some of the employed schemes include the integration of other smartphone sensors [140,141], the application of different filtering methods [142,143], and the use of machine learning approaches [144,145]. Another good future of the RSSI technique is that it can be applied not only for Bluetooth technology-based systems but also in other wireless technologies such as Wi-Fi [146], Bluetooth [86], and RFID [147,148].

#### 2.5.2. GPS-Based Proximity Estimation Technique in Contact Tracing Systems

The global positioning system (GPS) as was earlier described in Section 3.2 is a satellite-based localization system. The GPS estimates object position on the earth’s surface by the trilateration of the object with a minimum of three satellites.

**Figure 11:**
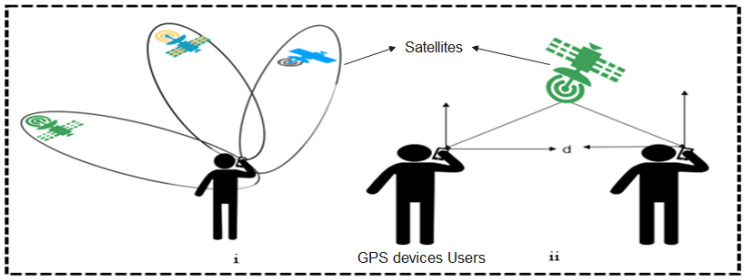
GPS-based proximity estimation using trilateration and triangulation satellite.

Trilateration is a localization technique that employs the distance of satellites whose positions are known to estimate the location of an object on the earth’s surface [149]. Since satellite broadcast their signals as a sphere, the intersection of the spheres from three satellites gives the exact location of the object as shown in Figure 11 (panel i). Another popular localization technique is triangulation. In triangulation, angles are utilized to estimate the location of an object as depicted in Figure 11 (panel ii).

However, GPS-based proximity estimation methods are susceptible to poor proximity estimation precision occasioned by factors such as signal delay in the space, blockage of satellite view by poor weather conditions, etc. Most importantly, GPS-based systems are not suitable for indoor applications [60,61,62]. Consequently, studies in contact tracing systems adopt hybrid approaches in their designs. For example, Banerjee et al. [150] proposed a hybrid contact tracing system by combining GPS and BLE data. The study proposes proximal, a graph-based contact tracing solution that seeks to achieve power efficiency and accuracy. The system when evaluated outperformed older systems by achieving 94% in both precision, sensitivity, and F-score. Similarly, Xiong et al. [151] also proposed a hybrid of GPS and BLE termed REACT. The novelty of this framework is that it assigns geoids to locations depending on the different risk levels. This provides the users with the ability to control the access level to their private details. When evaluated, the framework outperformed other similar frameworks. However, even though the hybrid solutions perform can be applied both in indoor and outdoor scenarios, the location privacy issues associated with GPS systems remain a challenge.

### 2.6. Privacy/Security Loopholes in Contact Tracing Systems

**Figure 12:**
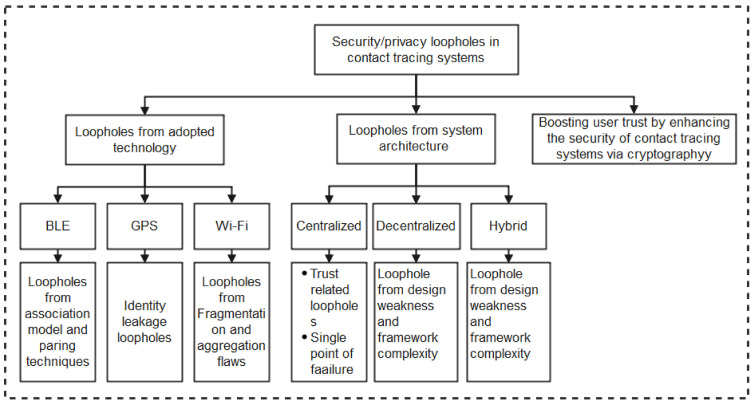
Security/privacy loopholes in contact tracing and social distancing systems.

The security and privacy of contact tracing systems play a major role in boosting users’ confidence. Consequently, a secure and privacy-oriented device will command a higher adoption rate thereby making the system more effective. We have identified as shown in Figure 12 that two major sources of security/privacy loopholes in contact tracing systems include: (a) adopted technology and (b) system architecture.

#### 2.6.1. Privacy/Security Loopholes due to Choice of the Technology

Material selection is a very important step in system design. This is because each technology has its peculiar security/privacy vulnerabilities. Various factors such as material availability, cost, and other constraints have continued to influence designers’ choice of materials. Unfortunately, where weak materials are selected, there is a corresponding privacy/security cost on the final product. In this sub-section, we examine the security vulnerability of some of the common technologies applied in contact tracing systems such as BLE, GPS, and W-Fi.

##### Security/Privacy Vulnerabilities of BLEs

There are three main association models for BLE devices. Starting from the weakest, they are (a) Just works (b) Passkey and (c) Out of band models. When two BLE devices (particularly versions 4.0 and 4.1) try to connect, they do so using a pairing method called the LE legacy pairing technique. This implies that after advertisement, the devices exchange temporary keys (TK) (and its extension called short term keys (STK) whose values depend on the association model adopted. For instance, systems that employ the just works association models set the TK to zero making its paring method the weakest. On the other hand, systems using the passkey model apply only a portion of the TK in their security. Consequently, the security codes are a few bits long, hence are guessable. Lastly, systems using the out-of-band association model apply the complete 128 bits TK for its security. However, their security codes are in plain text format which also leaves the system porous. Thus, it can be deduced that the privacy of BLE devices especially versions 4.0 and 4.1 which are the common BLEs in the market can easily be compromised through various attacks such as passive eavesdropping, man in the middle, relay, and denial of service attacks, etc. [152]. These attacks may lead to grave consequences such as adversaries gaining unauthorized access to the system to either obtain confidential/sensitive information, hampering the network performance, or even controlling the network remotely [153]. Fortunately, the later versions of BLE such as versions 4.2 and 5 connect via a more secure paring technique known as LE secure. In this paring technique, a long-term key is generated and encrypted using Elliptic Curve Diffie-Hellman (ECDH). Therefore, they are less vulnerable to privacy/security compromises. To further strengthen the security, when these higher versions are used, weak association models such as just works and pass-key models should be avoided, proper encryption mechanism should be adopted, source code should not be made public to avoid reverse engineering, authentication passwords should be made very strong, etc. [154].

##### Security/Privacy Vulnerabilities of GPS

GPS devices are majorly for sensing object locations with a time stamp. This implies that GPS-based contact tracing systems are likely to reveal the location and time of the user encounters [155]. Therefore, the main privacy vulnerability of GPS-based systems is the possibility of inferring the identity of users based on the location and time of encounters [156]. Consequently, from the GPS-based contact tracing data, it is possible to determine infected persons or has infected other people. Accordingly, unhealthy behavior such as stigmatization and hatred against some members of society may set in. Therefore, when GPS sensors must be used for contact tracing systems, efforts must be made to conceal the GPS data. For instance, GPS data can be encrypted so that an adversary may find them useless.

##### Wi-Fi Security/Privacy Vulnerabilities

Wi-Fi protocols are known to be susceptible to various attacks such as man in the middle, key recovery, traffic description, and denial of service [157]. It has also been discovered that there are some security flaws in Wi-Fi-based systems originating from the design of the IEEE 802.11 standard. These flaws are in frame fragmentation and aggregation functionality of all versions of Wi-Fi including wireless equivalent privacy (WEP) and the various versions of Wi-Fi protected access (WPA) such as WPA2 and WPA3 [158]. These flaws if exploited by adversaries could enable the gain unauthorized access to the Wi-Fi networks with the view of retrieving confidential information or harming the network. Moreover, security experts warned that this vulnerability is worsened if the Wi-Fi network is poorly configured. By implication, gaining unauthorized access to Wi-Fi networks is not easily achieved if the necessary security codes are configured in the network. Therefore, to minimize the impact of frame fragmentation and aggregation flaws in contact tracing systems, developers of such systems should pay serious attention to security configuration.

#### 2.6.2. Security and Privacy Loopholes due to System Architecture

As earlier explained, contact tracing systems are designed using either centralized, decentralized, or hybrid architecture. However, there is a correlation between the architecture and the security vulnerability of the systems. For instance, centralized systems are known to be susceptible to trust-related vulnerability. The handlers of the central database could for some reason decide to compromise the user privacy. Some possible reasons could be for national interests especially the foreign hosting companies and financial gratification such as sales of sensitive data. Furthermore, central systems suffer from a single point of failure. Once an adversary successfully gains unauthorized access to the central system, there is a total collapse of the privacy of the system [159]. On the contrary, the decentralized systems are not by a single central system, hence are not susceptible to centrality vulnerability. Therefore, research efforts should be directed towards developing robust decentralized contact training systems since it has been discovered that they are not vulnerable to centrality-related attacks.

#### 2.6.3. Boosting User Trust by Enhancing the Security of Contact Tracing Systems via Cryptography

Cryptography is the science of encoding messages in a manner that only intended recipients can access or understand the messages [160,161]. It is intended to achieve data security goals such as confidentiality, integrity, authentication, and non-repudiation, etc. The most popular technique of cryptography is encryption. Encryption is the process of securing information using secret codes. The two types of encryption are symmetrical and asymmetrical encryption methods. Symmetrical encryption (also called private-key) implies that both the sender and the receiver share the same secrete key. On the other hand, asymmetric encryption (also referred to as public-key encryption) requires two types of keys namely, public and private keys. The public key is available and can be seen by any person while the private key is exclusively for the authorized recipient [161].

Cryptography is applied in many real-life scenarios to achieve secure communication and end-end encryption in critical sectors such as but not limited to internet banking [162], vehicular ad-hoc network (VANET) [163], and web security [164]. Cryptographic algorithms are also applied in energy constraint devices such as the Internet of things (IoT) [165] and electronic health systems [166]. Nevertheless, the advent of COVID-19 has further increased the demand for e-health facilities.

COVID-19 came along with it a lot of myths and conspiracy theories that brought so much distrust against the global health professionals and the authorities of the various countries [167]. In this scenario, systems that cannot provide timely and accurate information in a privacy-preserving manner will lose users’ trust. Therefore, it is obvious that there exists a strong link between system security and users’ trust. Consequently, the concept of trust is very crucial in the life cycle of e-health solutions such as contact tracing systems. Since the system adoption rate is also tied to user trust, enhancing user trust in the system is critical. We, therefore, recommend that the same way trust prediction is performed for online users to determine their online reputation [168,169], trust prediction for e-health systems is also important. Because the system adoption rate (which is a function of user trust) is critical in determining the effectiveness of such a system, the importance of enhancing system security via cryptography cannot be over-emphasized.

Some security mechanisms based on cryptography have been proposed in this regard. For example, Sing and Raskar [167] proposed a privacy-preserving COVID-19 result verification framework. This protocol is aimed at reducing the spread of the diseases by providing a platform that, in a timely manner, verifies and grants access to only those who meet up with set rules. Some proposed contact tracing frameworks rely on data encryption. Similarly, An et al. [170] proposed a privacy-oriented technique for epidemic contact tracing (PROTECT). This protocol employs Brakerski/Fan-Vercauteren homomorphic encryption to achieve secure contact tracing. In addition, a privacy-preserving system named GoCoronaGo was proposed in [171]. This system applied asymmetric encryption where the public keys are displayed online while the private keys are issued offline. Furthermore, Kim et al. [172] proposed Safe contact tracing for COVID-19, a system that employs a functional encryption technique and optimization to achieve privacy-preserving contact tracing in addition to the visualization feature of the framework.

Moreover, eliminating trust bottlenecks has been identified as a new way to go. In this regard, BlockChain technology has been employed to achieve a trustless framework. In their recent research, Simmhan et al. [173] proposed a framework that leverages BlockChain technology and its cryptographic-based security to address trust-related issues in the COVID-19 use case. Other blockchain-based contact tracing systems such as [109,121] are already discussed in Section 2.4.7.

However, despite these efforts, a lot of research efforts are still required to achieve secure and privacy-preserving contact tracing systems.

## 3. Social Distancing Methods against COVID-19

The early stage of infectious diseases outbreaks is mostly characterized by the lack of vaccines or permanent cures for such diseases. In some cases, such as the COVID-19, vaccines may not provide the needed protection, hence, the need for a supportive measure. In line with this, the world health organization has advised that people should keep at least 2 m (6 feet) distance away from each other to be safe from the coronavirus [174]. Social distancing, therefore, is a non-pharmaceutical infectious diseases control and management strategy employed to reduce human interactions that may lead to physical contact during a pandemic situation [5,175].

**Figure 13:**
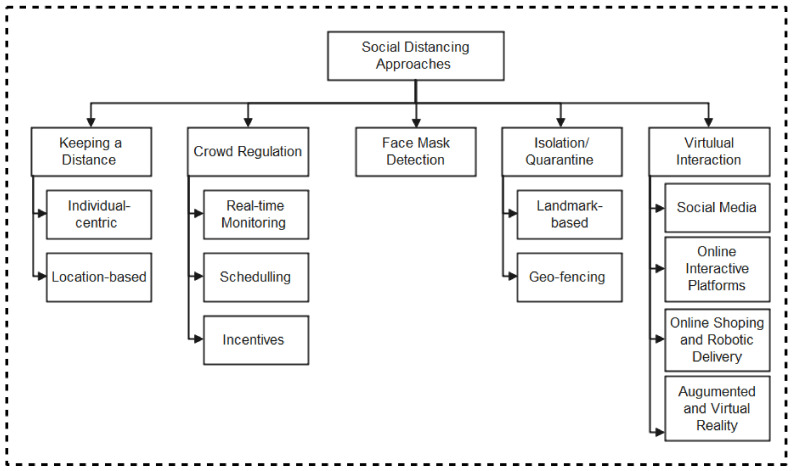
Approaches and Technologies for social distancing.

Moreover, the state-of-the-art approaches of social distancing are based on policy statements by government authorities. Such measures are usually enforced using approaches such as public place closures, placing a ban on public gatherings and events such as funerals, wedding ceremonies, etc. [7]. However, these policy-based approaches do not only cause discomfort to the people but also negatively affect their source of livelihood and the national economy at large. As a result, people find it extremely difficult to adhere to the social distancing protocols, hence, the need for assistive technologies. To alleviate the pains on the people, a lot of efforts have been expended trying to integrate technologies in social distancing. We have identified the four measures as shown in Figure 13 through which technologies may be applied to achieve effective social distancing. These measures include keeping a distance, crowd regulation, wearing of facemask, isolation/quarantine, and virtual interactions. We, therefore, discuss these measures and the respective technologies for achieving them.

### 3.1. Keeping a Distance

We have identified two scenarios where technologies can be deployed to help humans to keep social distance. They are either individual-centric or location-based technologies for keeping a distance to achieve social distancing. The individual-centric social distancing system assumes that every individual subscribes to the system and is always moving around with their devices. The devices are characterized by mobility to guide and guard the user even while on the move. On the other hand, location-based systems are designed to limit the spread of infectious diseases in designated places such as workplaces, event halls, markets, schools, worship centers, etc. We, therefore, classify social distancing into mobile and location-based social distancing scenarios. In this sub-section, we briefly discuss the technologies employed in each of these scenarios.

#### 3.1.1. Individual-Centric Measures of Maintaining Social Distancing

Personal protection is a key component of social distancing. Considering human mobility nature, some researchers have proposed social distancing frameworks that move along with the users. We classify such mobile social distancing devices and apps as individual-centric systems because it is expected that every individual should acquire them and wear or move along with them. Fortunately, smartphones which have become the closest companion to humans have become useful in the development of mobile social distancing systems. Another popular individual-centric social distancing implementation platform is the wearables such as bracelets, bangles, facemasks, etc. Since most existing individual-based social distancing systems are mostly powered by BLE, Wi-Fi, and a few other sensors such as passive infrared and ultrasonic sensors, we will dwell more on those technologies.

- BLE for individual-based social distancing

BLE has not only gained popularity in the fight against COVID-19 but is a choice protocol for mobile applications. This is because BLEs have endearing features such as low energy consumption, ease of deployment and can be applied for both indoor and outdoor environments. Furthermore, smartphones, where most COVID-19 preventive apps are installed already, have Bluetooth fitted by default. This implies that BLE is not restricted by location, hence can be applied in mobile scenarios.

Consequently, different studies have been carried out in BLE-based social distancing solutions especially for mobile scenarios. For example, Munir et al. [176] proposed a two algorithms model for social distancing. The first algorithm calculates the distances between two persons using the Bluetooth RSSI values. The algorithm also classifies the distances into different risk levels before recommending that the individual is safe or at the tail-risk using a probabilistic linear model. The second algorithm applies a curve-fitting model to perform risk optimization by creating a risk zone that guides the user in keeping social distancing. A prototype was implemented in an android environment and the result of the experiment shows that at 95% of conditional value-at-risk (CVaR) confidence, the model can handle 45.11% of the risks associated with the user’s safe distance estimation.

Similarly, Kumar et al. [177] presented a BLE-based social distancing scheme that monitors users and warns them when too close to other users. The system also has three levels of feedback namely: green, which implies that the user is at safe distance but needs to be cautious. The next color code is yellow which implies a warning. Finally, the red color signifies danger. An evaluation of the framework on different android phones showed a variation of ±2 dBm in signal strength.

Moreover, another BLE-based social distancing study was conducted by [178]. In this work, 1612 data points were analyzed statistically and observed that an RSSI value of less than −48 dBm bridges social distancing threshold or otherwise. A simple neural network was applied in data classification and further analysis. The evaluation of this framework achieved an accuracy of 89.9%. This study however failed to consider phone orientations when held at different places such as chest pocket, trouser pocket, held in hands, etc., different phone models. The study also did experiment with different phone models to see if there could be a variation in the RSSI values.

Furthermore, Rusli et al. [179] proposed My safe distancing (MySD) which utilized a BLE signal for proximity detection and sends an alarm if the set distance threshold is violated. The system is continuous until the required spacing is achieved. The system adopts Government classifications of locations as safe (zero cases), unsafe (few cases), and danger zone (many cases). Google map and GPS localization are applied to determine the user’s location to warn if in an unsafe or dangerous zone.

- Sensor for Individual-Based Social Distancing

Sensors are embedded in various devices and can detect environmental changes and make a decision based on these changes or communicate collected data to other devices designated for decision making. Sensor-based methods perform their sensing by converting environmental quantities such as voltage, pressure, temperature, humidity, the pressure of gases to electrical signals. Examples of sensors include motion sensors such as accelerometers, gyroscopes, and magnetometer sensors [180]. Their applications span different fields of human endeavors such as military surveillance, industrial applications, environmental monitoring, and other medical application areas [181]. In addition, sensors are widely applied in the detection of objects such as humans, vehicles, social distancing, and other targets of interest.

In the social distancing scenarios, sensors have been considered a veritable tool for mobile applications. These sensors are applied mostly in wearable systems. For example, a wearable social distancing solution using an oscillating magnetic field sensor was proposed in [109]. This framework employs two magnetic coils which include a 20 kHz magnetic field transmitter and a magnetic field receiver. The developed prototype not only conforms with the user privacy protocols as is being promoted by Apple–Google [35] but also overcomes the multipath propagation errors which are common in existing wireless systems. However, the prototype needs further miniaturization required of contemporary wearables.

Furthermore, a social distancing smart cap called Suraksha that uses passive infrared sensors (PIR) was proposed in [182]. The sensors monitor the environment and alert the user once the social distancing protocol is bridged. The system is comprised of three passive infrared (PIR) sensors carefully positioned to cover a 360° area view and a range of 1.5 m. However, the sensors’ alignment may shift during usage thereby reducing the system sensing window.

Moreover, an ultrasonic sensor-based social distancing system was proposed in [183]. The system employed ultrasonic HC-SR04 and a microcontroller Arduino Nano to develop a smart ID card for social distancing. Similarly, another ultrasonic sensor-based social distancing scheme is also presented in [184]. The study proposed an ARM microcontroller and ultrasonic sensor for the development of a wearable social distancing system with an inbuilt LCD user interface. However, although this concept gave insight into sensor-based human proximity sensing, the choice of ultrasonic sensors whose sensing window is about 20° may lead to false-negative errors (where the user is bridging the social distancing protocol but the system fails to detect it). Furthermore, ultrasonic sensors are sensitive to both animate and inanimate objects [185]. This may as well lead to false-positive errors (falsely alerting the user when close to non-human objects.

- Hybrid technology for keeping a distance

Some studies proposed a combination of two wireless technologies to get a robust social distancing solution. For example, ref. [186] proposed the combination of BLE and UWB for social distancing. The combination of these two wireless technologies is aimed at compensating for each other’s weaknesses. The BLE is known for poor accuracy while UWB has relatively high sensing accuracy. On the other hand, while BLE is more energy-efficient, UWB is more energy-demanding.

Another proposal that combined Ultrasound and BLE is reported in [187] where proximity estimation was calculated using time of flight (TOF). The proposed system is not only distributed but operates in the background in an energy-efficient manner. However, it is worth mentioning that for commercial deployment, ultra-sound systems are both expensive and complex to implement. In summary, we have presented the features of the individual-centric approach to keeping a distance in Table 8.

Moreover, despite the various applications of wireless networks, there are various security vulnerabilities that designers of social distancing systems via wireless signals need to plan against. Some of the possible attacks of wireless networks are denial of service attack, sniffing attack, man-in-the-middle attack, eve dropping attack, etc. [188].

#### 3.1.2. Location-Based Approaches for Maintaining Social Distancing

**Figure 14:**
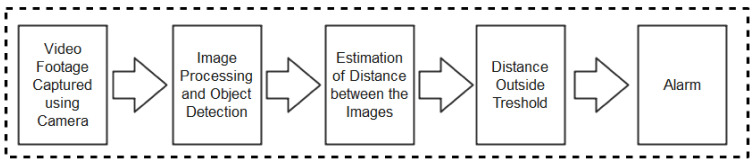
Procedures for social distancing via computer vision.

Location-based approaches to keeping a distance describe those systems deployed to ensure that people who visit such locations keep to social distancing rules. The systems are designed to monitor the locations and ensure that each person is at least 2 m farther from the other. Once a breach of this protocol is detected, a warning alarm is sent to the offender. Various studies have been reported in location-based approaches to maintain social distancing. However, it has been observed that computer vision approaches are still the most popular location-based proximity detection technique [189,190]. Computer vision is a computing knowledge area that aims at mimicking human vision. This entails the use of visual sensors (cameras) for the acquisition of videos and images for processing and analysis to extract meaningful numerical or graphic data that can be interpreted for decision making [191]. One key application area of computer vision is in object detection which is the identification and location of objects in an image or a video [152,153]. Proximity estimation is an extension of object detection which performs an additional task of computing the respective distances between the objects and alerts the offenders as shown in Figure 14.

Image processing in computer vision has been enhanced by deep learning techniques such as Yolo, Faster R-CNN, Single Shot Detector (SSD), and Region-based Fully Convolutional Networks (R-FCNN), etc. [192]. To achieve location-based social distancing, many computer vision and deep learning solutions have been proposed. For example, ref. [193] proposed Deep SOCIAL, a CCTV-based social distancing solution with the combination of computer vision and YOLOV4 deep neural network for people detection in both indoor and outdoor environments. The study incorporated adapted inverse perspective mapping (IPM) technique and simple online real-time tracking (SORT) algorithm into the DNN framework for proximity estimation and object tracking respectively. When evaluated with Microsoft Common Objects in Context (MS COCO) and Google Open image datasets, the study achieved pedestrian detection accuracy of 99.8%.

An attempt to overcome the privacy vulnerability of computer vision-based methods of object detection was presented in [194]. In this study, monocular cameras were proposed for real-time human image detection. The major enhancement in this work is that, unlike the conventional computer vision approaches that record the videos or images using traditional cameras, this study proposes monocular cameras for real-time image detection and analysis, hence leaving no trail that could form the basis for privacy infringement. To evaluate the framework, two pre-trained deep learning algorithms including Faster R-CNN and YOLOv4 were pre-trained and employed for the image processing and analysis to determine if the persons whose images are analyzed have violated the social distancing rule or not. Where a violation is detected, an audio-visual warning is sent to encourage people in the location to readjust. Nevertheless, there is a need to enhance this framework so at to overcome occlusion at high-density pedestrians and accommodate some factors that may affect pedestrian classifications such as groups and close relationships.

Moreover, Neelavathy et al. [195] presented a simple computer vision-based social distancing enforcement system termed Smart Social Distancing (SSD) mobile application. In this work, smartphone cameras were used to obtain the video footage of people. Human image detection in the video was achieved using YOLOV4 deep learning image processing algorithms while the proximity estimation between the persons for social distancing protocols violation detection was performed using the Euclidean method. The study also incorporates a Bluetooth technology option where proximity estimation is performed using BLE received signal strength (RSSI). However, the study used a mobile phone camera that has a limited view area. Furthermore, the only android-based app was developed which limits the to only android phone users.

Furthermore, a privacy-preserving computer vision-based social distancing framework was reported in [196]. This study aims at achieving a cost-effective social distancing framework that employs a neural network to detect humans using either fixed or mobile cameras and does not rely on ground plane estimation. Similarly, Punn et al. [197] proposed a system that utilized YOLOV3 for object detection and a Pair-wise vectorized approach for proximity detection. Moreover, notwithstanding the popularity of computer vision in location-based social distancing, object detection accuracy is still a challenging factor. Although computer vision-based approaches are popular and accurate, there are some weaknesses associated with them. To start with, video or photo quality determines the efficiency of image recognition and tracking. This implies that the system efficiency may drop in a dark environment since the images will likely be blurred. Again, the computer vision approach of object detection still has some challenges such as high installation cost, complexity in video analysis and processing, and privacy-related concerns [198]. A summary of location-based social distancing using computer vision is presented in Table 9.

### 3.2. Crowd Regulation Measures

**Figure 15:**
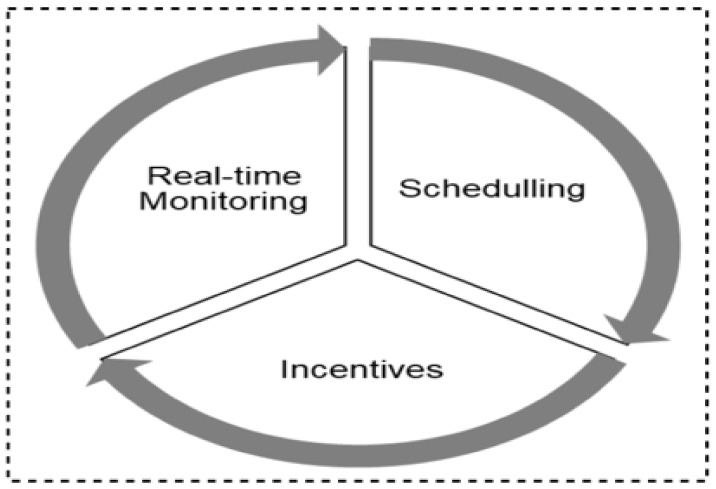
Measures for crowd regulation for social distancing.

The second approach to achieving social distancing against infectious diseases is to regulate entry into public places. There are three major measures as shown in Figure 15 to regulate the crowd for social distancing purposes.

#### 3.2.1. Real-Time Monitoring

**Figure 16:**
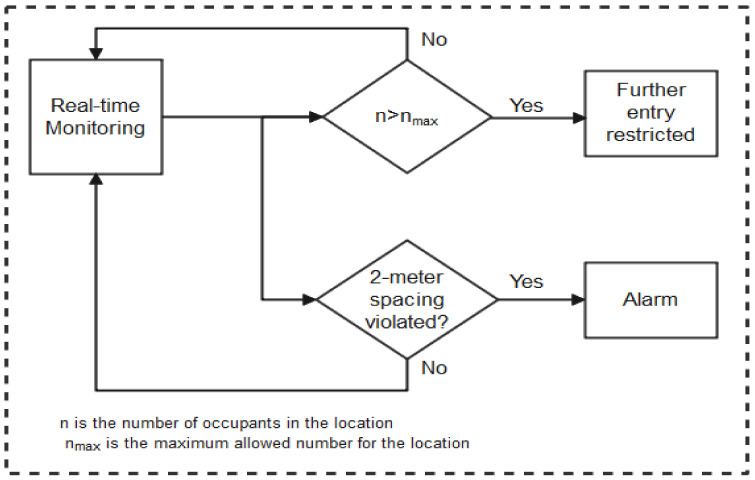
Real-time monitoring strategies for social distancing.

Real-time monitoring systems are automated crowd regulation solutions for social distancing, especially in public places. As shown in Figure 16, real-time monitoring systems are designed to enforce social distancing by employing either of the two key strategies or both. The first strategy is to regulate the population of people in public locations by ensuring that the number of occupants is within a given threshold which if exceeded, further entry is restricted. This implies that real-time monitoring systems continually perform the head count of the occupants to ensure the allowed number is not exceeded. The second strategy is the monitoring of human–human spacing in the location to avoid close contacts that may lead to the transfer of the COVID-19 virus from one person to the other.

Various technologies have been employed for real-time monitoring of public places for social distancing. Two popular technologies employed for real-time monitoring are Wi-Fi and sensors/IoTs.

- *Wi-Fi for Real-time monitoring*: Wi-Fi-based solutions utilize users’ smartphone Wi-Fi signals for population determination and distance estimation. For instance, a Wi-Fi-based crowd control system for regulating crowds in shopping mall settings is proposed in [199]. The system performs digital counting of shoppers in a shopping mall thereby detecting when the mall is overcrowded or otherwise. The security guards utilize the information provided by the system to regulate entry into the mall for social distancing. The system also triggers an alarm when the occupancy level exceeds a certain number alerting each shopper to keep a certain distance from others. There is also a web-based component of the system through which the general public can know the status of the mall ahead of time to know the appropriate time to shop.

Similarly, Oransirikul et al. [200] proposed a Wi-Fi-based solution to control human congestion in public transportation terminals. The system monitors Wi-Fi signals from people’s mobile devices to determine if the number of persons in the terminal is still within the threshold, otherwise, an alarm will notify people to spread from each other.

Furthermore, Wi-Fi/IoT-based system called SafeMobilility which monitors congestion in an indoor location was presented in [201]. The system performs proximity sensing and warns occupants when the threshold of 2 m is violated. Furthermore, the system determines people’s location, counts the occupants, and sends an alarm when the capacity of the location is exceeded. When evaluated, the system achieved 91% accuracy positioning.

However, one limitation of Wi-Fi-based real-time monitoring systems is that some users may turn off the Wi-Fi on their mobile devices. If this happens, the system will be blind to occupants whose devices’ Wi-Fi is turned off. Consequently, the headcount will not reflect the true number of persons in the location and the distance estimation will also be faulty.

- *Sensors for Real-time monitoring***:** In this approach, various proximity and vision sensors are integrated into wearables, robots, or other smart devices to enforce social distancing. These sensor-based systems perform proximity estimation and issue a warning alarm to users if social distancing rules are violated.

Various studies have been carried out in this area. For example, ref. [202] presented a sensor-based framework for social distancing detection, monitoring, and enforcement. In this framework, a robot is fitted with multiple sensors including an RGB-D camera and 2-D light detection and ranging (LIDAR). The robot moves around and tries to measure the distance between people in the crowd with the help of the sensors. Once a violation of the social distancing rule of 2 m apart is detected, a message is displayed on the robot’s screen which encourages the persons involved to disperse and keep the required distances apart. The robot is also fitted with thermal temperature measurement for remote body temperature monitoring by health workers. An experimental demonstration showed that the framework performed better when integrated with CCTV. Nevertheless, there is a need to improve on the method of communicating with violators. Furthermore, an algorithm is needed for the classification of human targets to differentiate their relationships.

Likewise, ref. [203] demonstrated that existing infrastructures in smart environments can be utilized to enforce social distancing. The study performed an extensive analysis of sensor data between 2017 and 2018 in a collaborative indoor smart space and was able to give insight into the location occupancy level, occupancy pattern, and potentially transmission level. From the analysis, both safe and crowded locations can be identified so that people will be advised to occupy safer locations.

Similarly, an indoor crowd control system that allocates people within an indoor environment was proposed in [204]. The study aims to enforce social distancing among people in an indoor location by allocating people appropriately without displacing those already occupying the location. The two evolutionary optimization algorithms namely, particle swarm optimization (PSO) and ant colony optimization (ACO) were employed. When evaluated benchmarking other approaches such as random placement of nodes and the generic algorithm (GA), the system performed better.

Furthermore, ref. [205] proposed a vision sensor-based framework for enforcing social distancing. This study proposed a framework that employed artificial intelligence to analyze monocular camera data to detect social distancing violations in real-time before issuing audio-visual warnings where applicable. Although there were some missing detections due to high pedestrian density and occlusion, the framework is still effective as most of the pedestrians were detected.

Nevertheless, the many challenges associated with sensor-based social distancing schemes. Some of the challenges include sensor sensitivities, sensing coverage, and range, sensor network security, and system power management [206].

#### 3.2.2. Scheduling

Scheduling in the context of social distancing is a measure aimed at arranging access into or out of a location to avoid crowds, especially in the pandemic period. In a pandemic period such as the COVID-19, many people patronize public places such as hospitals, schools, and shopping centers. Some studies have been published on the various approaches to reduce crowds in public places. For instance, Garg et al. [207] proposed a Blockchain-based movement pass for citizens of a country. This will ensure that only those with an active pass can move to a designated place at a given time. By so doing, the population of people in public places will be regulated. Furthermore, people who have been diagnosed or believed to have closely interacted with infected persons will not be granted a pass.

Similarly, the University of California, San Francisco (UCSF) Health developed a COVID-19 self-triage and self-scheduling app aimed at optimizing hospital usage during the pandemic period. Although the system was an emergency intervention, it was very helpful to the extent that 1129 patients used the app within 16 days [208]. On the other hand, a decisional support system (DSS) that uses mixed integer programming (MIP) for health workers scheduling during the COVI-19 pandemic is presented by [209]. The system generates a roster with a balanced workload among workers in a manner that guarantees minimal exposure. Equally, mixed-integer linear programming (MILP) was also applied to develop a staff scheduling system for a pharmaceutical firm in Italy. This study aims to develop a system that allows a defined staff on duty without bridging staff contracts in terms of working hours. When evaluated, the system outperformed the one currently used in the firm [210].

#### 3.2.3. Incentives

As was advocated in [7], incentives such as compensations, monetary gifts, etc. could encourage people to obey social distancing rules. Correspondingly, Manoj et al. [211], proposed an incentive Blockchain-based database of people’s travel history aimed at early detection and informed decision on the likely status of an individual was proposed. The framework also advocates for a kind of compensation for those who adhere to the COVID-19 protocols such as self-isolation, social distancing, and voluntary test requests.

The strengths and weaknesses of some of the studies to regulate crowds are summarized in Table 10.

### 3.3. Isolation/Quarantine and Encroachment Prevention Technologies

Another key strategy in social distancing protocol is the separation of people who are exposed to or diagnosed with the infection from the rest of the society to avoid further spread. Quarantine is the withdrawal of the people who are exposed to the infection from the rest of the public. In such an instance, the exposed persons separate themselves from the rest of the society for a given period such as 14 days in the case of COVID-19. On the other hand, isolation refers to the mandatory restriction of patients who have been diagnosed with the infection in an environment where they will not mix up with the rest of society [212].

In both instances, there is a need to ensure maximum compliance from both the separated persons and the rest of the community especially friends and well-wishers. There are two major approaches to monitor isolation/quarantine which are geofencing and landmark-based solution center.

#### 3.3.1. Landmark-Based Isolation and Quarantine Systems

Landmark-based solutions are designed using a known structure or fixed object within the user’s residence. Users are expected to prove that they are within the landmark whenever necessary. For example, the Ukrainian Act at Home [213] is a public-owned self-isolating app linked to a user’s driver’s license and vehicle particulars. The app prompts the users at random times to take a snapshot showing their faces in their registered landmarks in the isolation center. The pictures are uploaded within a period of 15 minutes of being prompted to do so failure of which may be interpreted that the patient has wandered away from the isolation area. The app monitors those in mandatory isolation and ensures their movements are restricted within their isolation area.

Similarly, the home quarantine app of Poland [214] is a monitoring app that employs GPS and facial recognition to enforce self-isolation for both diagnosed and exposed persons. It is used by the authority to ensure that infected or risky people do not move beyond the quarantine/isolation area. This is achieved by letting users confirm their locations by taking and uploading pictures in their registered addresses within twenty minutes after being prompted to do so. The system also allows users to perform basic health checks and report emergencies to the relevant authorities.

#### 3.3.2. Geofencing for Isolation/Quarantine

One major measure to enhance isolation/quarantine is digital geo-fencing. This approach tries to monitor a location to avoid trespassing into/out of the restricted zone. A notable example is the Kyrgyzstan StopCovid-19 KG mentioned in [215]. This app was powered by the operational headquarters of the Kyrgyz Republic using some experts under the umbrella of the State Committee for Information Technology and Communications (ITC) in the Kyrgyz Republic. After the development, the app was donated to the health authority for the fight against coronavirus. It uses a GPS-based map for surveillance of areas occupied by people already infected by COVID-19 so as detect people who have closely interacted with them or prevent people from encroaching into the area. The app also helps to prevent influx into the Kyrgyz Republic to arrest the further spread of the dreaded COVID-19. However, there have been a series of security and privacy concerns against the StopCovid-19 PK app. First, users are not sure of which individual or organization is collecting the data, how the data is processed, and where the data is stored. Furthermore, there were reports of privacy breaches where users’ private data surfaced online. Finally, the app has been observed to show that users visited were in actual sense they never visited [216].

Another social distancing enforcement tool is the StayHomeSafe of Hong Kong [217]. This app which is integrated with a smart wrist bangle allows the user to create a digital perimeter fence representing the isolation zone. When a violation is suspected, alarms are sent expecting the user to perform a QR code scanning within 15 s. If the QR code scanning is performed within the prescribed 15 s, it means the user has not strayed beyond the confinement zone. Otherwise, it implies the user has violated the isolation rule of which the health authority is expected to take further actions.

### 3.4. Virtual Interactions

Virtual interaction is a phenomenon that allows people to relate in an online environment without having any physical contact. It became the alternative way of life for the people’s interactions following the ban on physical contact. This is possible because the virtual interaction concept has recreated the world and introduced some new concepts such as virtual communities and global villages which are not affected by geographical boundaries [218]. Consequently, there is a new way of teaching and learning, healthcare delivery, business meetings, in fact, a new way of doing everything [219]. The virtual interaction technologies on their own may not enforce social distancing, but they provide platforms that keep human activities going thereby encouraging. They mimic physical interaction scenarios by digitally bringing different people who are physically located in different parts of the world to interact in real-time. In other words, virtual interaction technologies encourage social distancing by creating a digital world for people to interact as if they are physically located in the same environment. Some virtual interaction technologies which played a major role in the fight against COVID-19 include social media applications, web conferencing and distance learning platforms, and online shopping and drone-based home delivery systems. The features of existing virtual interaction platforms for social distancing are presented in Table 11.

#### 3.4.1. Social Media Apps

These are the interactive online environments that enable people to share multimedia files, plain text messages, ideas, and other information among members themselves. Some examples of social media platforms include Facebook, Twitter, WhatsApp, Telegram, YouTube, etc. [220]. Social media apps have been useful tools that helped people during the hash and compulsory social distancing policy that led to lockdown in many countries. Social media became the main source of interaction among people hence the sudden increase in its usage within the peak COVID-19 period [221]. It has also been opined that social media has helped in social distancing. For instance, ref. [222] showed how social media became a tool that connects the teachers and the students and fostered active learning since the period when face-to-face contact became unsafe. Moreover, studies in [220,223] reveal that social media users promoted social distancing protocols. This led to mass acceptance of social distancing in most parts of the world irrespective of its social-economic adverse effects. However, because social media is open to all, professionals and non-professionals alike, and is not regulated in many countries, information received from social media may not be fully relied upon as being accurate [224,225].

#### 3.4.2. Online Interactive Platforms

**Figure 17:**
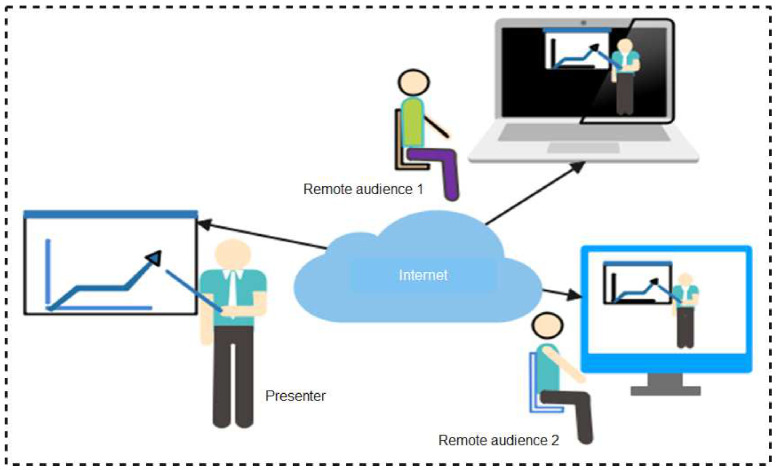
Online interactive platform.

Online interactive platforms are systems that bring people from different locations of the world into a virtual environment for real-time interaction as shown in Figure 17. Two major examples of online interactive platforms are web-conferencing and distant learning systems. The web conferencing apps also referred to as video meeting or online meeting apps are internet-based real-time interactive platforms that connect people for purposes of discussions, presentations, teaching and learning, meetings, and multimedia data sharing irrespective of their geographical locations. Some popular web conferencing platforms include Zoom, GoToMeeting, Cisco WebEx, and Google Meet, Zoho meeting, Microsoft teams [226,227,228]. Despite that web conferencing platforms have been in use over a long period, the outbreak of COVID-19 coupled with its consequent social distancing protocols have caused unprecedented multiplication in their usage especially in the field of education.

Similarly, distance learning platforms, even though they may not be fully equated to face-to-face learning, are very helpful in keeping the light of education burning in a socially distancing compliant manner [229]. Some of the popular distance learning platforms are Coursera, Skillshare, Udacity, Udemy, Edx, and Future Learn. Indeed, web conferencing and distance learning platforms brought succor to the education sector because they provided a virtual environment for academic activities such as conferences, symposia, lectures, and presentations that would not have been possible due to the social distancing rules. To say the least, these systems contributed immensely to the enforcement of social distancing protocol imposed on humans by the dreaded coronavirus.

#### 3.4.3. Online Shopping Apps and Robotic Home Delivery Services

Online shopping apps are web-based systems that provide people the platforms to buy and sell goods and services over the internet [230]. This has become a viable alternative to conventional shopping where people physically visit the malls or the market for daily purchases of goods and services. The COVID-19 pandemic which brought about social distancing has forbidden people from freely moving about for their daily activities including shopping in the conventional malls and markets. This posed serious hardship to the society which would have forced people to revolt against the government. Moreover, patronage of online shopping platforms was recommended to reduce the difficulties associated with social distancing [231].

Consequently, the patronage of online shopping services increased during the social distancing era in many countries such as Qatar [232], Germany [233], and Taiwan [234]. There are many online shopping platforms in different countries. For instance, in Nigeria, popular online shopping sites include Jumia.com.ng, konga.com, slot.ng, OLX.com.ng, etc. [235]. To further enhance social distancing by reducing human contacts, drones were being employed for the in-home delivery of goods and services. These innovative drone-based home delivery services have positively reshaped people’s perceptions of the social distancing protocol. In the United States, drone delivery has been reported to be very beneficial [236]. In the fight against coronavirus, the study in [190] proposes a drone-based delivery service for the tourism and hospitality industry. The drone-based services are aimed at uniquely minimizing human contact, hence, encouraging social distancing against the coronavirus.

#### 3.4.4. Augmented and Virtual Reality

Virtual reality (VR) technologies launch the user into a computer-generated 3D simulated environment giving an impression of a different location entirely. In other words, VR projects the user into an artificial environment by taking over some or all the five senses during the interactions. Some virtual reality systems include 360° videos and Oculus Quest headset and the Pokemon go game [237]. Virtual reality tools can be applied in scenarios like virtual tours, meetings, and conferences. Other application areas include educational, medical, and military training. Furthermore, it has been noted that virtual reality promotes teamwork and a sense of togetherness without incurring the cost of traveling. Experts have opined that VR is a very useful tool in combating COVID-19 since it can effectively reduce people’s physical contacts yet provides an environment that appears as though the people are in their original interactions [197,198]. VR can be applied in the fight against COVID-19 especially in telemedicine, awareness campaign, and other activities that enhance the efficiency of health services such as medical training during the lockdown period. VR was indeed very useful in medical training to the extent that the authors recommended its real-life implementations [240].

Similarly, augmented reality (AR) was also applied in social distancing against COVID-19. Unlike VR which launches the user into a new digital realm, AR only adds some audio-visual and other digital enhancements to the user’s interactions with the real-world environment [241]. AR has been proposed as pro-social distancing technology in many scenarios. For instance, ref. [242] has opined that the tourism sector can be revived through the application of AR. It has also been shown that financial managers and auditors can maintain social distancing and reduce travel costs by leveraging AR technologies for the effective discharge of their duties [242]. A review presented by Vuta et al. [243] shows that AR has been extensively considered as a tool that can re-launch the global education sector without violating social distancing protocols.

### 3.5. Facemask Detection

The wearing of a facemask is a key strategy in flattening the infection curve of the COVID-19 virus. Before COVID-19, not much research efforts were expended on facemask detection. However, the outbreak of the COVID-19 pandemic necessitated social distancing and mask-wearing thereby attracting so much research attention in this area of study [244]. Similar to human proximity detection, facemask detection has been performed mainly by artificial intelligence techniques such as convolutional neural networks (CNN) and deep learning models.

For example, a facemask detection model was presented in [82,245] where a combination of different super-resolution and classification networks (SRCNet) for mask detection and classification was proposed. The model utilized 3835 images from the medical mask dataset of which 671 were unmasked, 134 incorrectly worn masks, and 3030 correctly worn masks. The evaluation result shows that the framework achieved 98.7% detection accuracy. Additionally, a deep learning network model of mask detection known as Facemasknet was proposed in [246]. In their study, 35 numbers of image datasets comprising 10 unmasked images, 10 correctly worn masks, and 15 incorrectly worn masks were used to train the system before integrating it into the mask detection algorithm. The evaluation shows that the system achieved 98.6% mask detection accuracy. However, it could be seen that the training dataset is too limited, hence the system may not perform effectively in real-life scenarios.

Furthermore, a CNN-based study that employed a combination of Keras/TensorFlow and OpenCV was proposed in [247]. The authors had 1376 images—690 with mask, 686 no mask. A total of 560 images were later selected which 80% was used as training images and the remaining 20% was reserved as testing data. After evaluation, accuracies of 98.86% were achieved for training data, 96.19% for testing data, and 96% as the overall accuracy. However, the framework suffers delays in detection time.

Furthermore, Jiang et al. [248] proposed a Retina facemask that follows the object detection framework proposed in [109]. The framework likens object detection activities to a human body system that depends on the head, the neck, and the backbone for its functionality. The study uses ResNet or MobileNet as the backbone, Feature Pyramid Network (FPN) as the neck, and context attention modules as the head. A total of 7959 dataset images (with and without face masks) were trained and transferred to the mask detection system. Data filtering was performed using the object removal cross-class (ORCC) algorithm. During the evaluation, 2.3% and 1.5% greater than the baseline accuracies were achieved in face and mask detections respectively. However, even though a high accuracy was recorded, it was observed that not all the components of the system worked well with the ResNet backbone.

Moreover, a facemask detection framework named SSDMNV2 was proposed in [249]. This technique employs OpenCV deep neural network, TensorFlow, Keras, and MobileNetV2 architecture for image classification. The solution is cost-effective, lightweight, and showed an accuracy of 92.64%. Similarly, Pynq-YOLO-Net—a lightweight Convolutional Neural Network (CNN) and the YOLO object detection model-based facemask detection framework is proposed in [250]. Very large datasets from various sources including RMFD, MFDD, SMFRD, and MAFA were gathered and added into the training dataset for the development of a facemask detection system. After the training and integration into the system, upon evaluation, the detection accuracy of 97% was achieved. However, this proposal has not been evaluated in a real-life environment such as a CCTV camera.

Likewise, a Yolov2-based ResNet-50 deep learning model for mask detection is presented in [251]. In this study, 1415 images were extracted from the medical mast dataset (MMDS) and face masked dataset (FMD) and included in the training dataset. Mean intersection over union (MIOU) was used to enhance detection accuracy of which 81% was achieved using Adams optimizer. A related study that fine-tuned the Inception v3 deep learning model was proposed in [252]. Here, 1570 images were trained and tested in a simulated masked face dataset (SMDFD) applying image augmentation to take care of the limitedness of images. The model achieved 99.9–100% detection accuracy. Table 12 shows the features of some of the existing facemask detection technologies.

## 4. Discussion

The review work is a comprehensive study on the use of technologies as a non-clinical measure against COVID-19. The study specifically reviews digital methods of contact tracing and social distancing schemes for combating the coronavirus. In the study, contact tracing methods were reviewed under three sub-headings, namely, technologies, protocols, and apps.

Our review of the technologies in contact tracing systems shows that most contact tracing system developers prefer Bluetooth low energy (BLE) as the proximity sensing technology. This is because BLE is cost-effective since they are already fitted in most smart devices by the manufacturers. Other sensors such as GPS and Wi-Fi are also sometimes used in contact tracing systems as proximity sensing technology. However, although these other sensors are also available in many smart devices, BLE is more energy conserving [61] and flexible in framework designs, hence, is more user privacy conserving. In addition, despite its poor accuracy [253,254], most authors prefer the use of the received signal strength indicator (RSSI) as the proximity estimation technique in contact tracing systems. This might be because of its ease of implementation, especially with BLE-based solutions.

Moreover, smartphones are the most preferred implementation platform for contact tracing systems. The reason is that mobile phones have become an indispensable part of human lives. People move about with them; therefore, mobile phones can be used to track human locations cost-effectively. Nevertheless, smartphone devices are prone to sensing errors if positioned in a place where human muscle tissues or other obstacles can obstruct their signal line of sight or if positioned in different orientations or alignments such as putting them in back or chest pockets. Furthermore, being that some users place their smartphones inside other containers such as briefcases and handbags, it implies that readings taken from the smartphone’s sensors may not represent the accurate colocation distances [60,211].

Moreover, the study identified two major sources of security and privacy loopholes in contact tracing systems, namely loopholes due to adopted technologies and from chosen architecture. We discovered that sensing technologies such as BLE, GPS, and Wi-Fi have some inherent privacy and security vulnerabilities that system developers must consider during their designs to avert some security/privacy lapses that may lead to unauthorized access and compromise [152,155,157]. Secondly, there are loopholes in contact tracing systems due to the adopted network architecture. The study discovered that there is a correlation between the architecture and the security vulnerability of contact tracing systems. For instance, centralized systems are known to be susceptible to trust-related vulnerability. The handlers of the central database could for some reason decide to compromise the user privacy. Some possible reasons could be for national interests especially the foreign hosting companies and financial gratification such as sales of sensitive data. Furthermore, central systems suffer from a single point of failure. Once an adversary successfully gains unauthorized access to the central system, there is a total collapse of the privacy of the system [159]. On the other hand, the decentralized frameworks are complex both in design and system management but, they are not vulnerable to a single point of failure since the goal is to eliminate central control of the system.

On the part of Social Distancing, the study identified five major approaches of achieving social distancing, namely, keeping a distance, crowd control, face mask, isolation/quarantine, and virtual interactions.

It was observed that smartphones and wearable devices are mostly applied in the individual-centric method of keeping a distance. This is because individual-centric devices require mobility, hence need mobile tools such as smartphones and wearable devices. On the other hand, computer vision approaches are more popular in the location-based method of keeping a distance. This method requires the installation of cameras for capturing images and videos through which social distancing is enforced.

Moreover, the study identified two major approaches of enforcing isolation/quarantine of infected persons, namely, landmark-based and geofencing methods. Our review shows that both methods have been employed by some countries. For instance, the Ukrainian Act at Home [213] and the home quarantine app of Poland [214] are both landmark-based isolation/quarantine apps while the Kyrgyzstan StopCovid-19 KG and StayHomeSafe of Hong Kong are both are geo-fencing-based measures.

Likewise, the study outlines some virtual interactions platforms such as social media platforms, online interactive platforms, online shopping and robotic delivery services, and augmented and virtual reality. These platforms were very vital in encouraging social distancing [220,223]. One major strength of virtual systems is that information sharing is cost-effective since the platform is open to the general public. However, they are not regulated in many countries and hence could spread false information [224,225]. Furthermore, although virtual interaction systems played a very vital role in the education sector particularly during the COVID-19 when social distancing forbids facet-face classroom studies, it was opined that they are not natural and are not appealing to some people, hence may not effectively replace the classroom-based studies [229].

Finally, face mask detection is a major measure of enforcing social distancing. This strategy automatically discovers people who are not compliant and take the necessary actions required for enforcement in that location. However, our review has discovered that some frameworks were performed poorly due to some factors such as inadequate training data, poor detection accuracy, and poor evaluation methods.

Generally, a combination of contact tracing and social distancing schemes when properly implemented will go a long way to minimize the impact of contagious diseases. System efficiency and privacy of users are two key issues. However, there seems to be a dilemma of sacrificing user privacy for system efficiency and vice versa. This dichotomy has spurred a silent argument on one part that for the systems to be efficient in the task of contact tracing and social distancing, users should overlook their privacy vulnerabilities in the interest of saving lives. For instance, the BlueTrace and Pan-European Privacy-Preserving Proximity Tracing (PEPP-PT) protocols emphasized more on system efficiency than user privacy. On the other hand, decentralized protocols such as DP-3T, Apple–Google protocol have prioritized user privacy though not undermining system efficiency. This perspective advocates that user privacy conserving is a priority in health techs such as contact tracing and social distancing systems. There is therefore a need to strike a balance is to develop a protocol that will achieve both efficiency and user privacy preservation.

## 5. Open Research Issues

In this section, open research directions identified during this extensive review work are presented. This section highlights key areas where interested researchers and experts should channel more efforts to enhance the effectiveness of contact tracing and social distancing systems. The open research directions are therefore hereunder presented:

- *Architecture-based System Security and User Privacy Issues:* From our extensive review, we have discovered that system security and user privacy have continued to pose serious challenges to contact tracing and social distancing systems. We have also discovered that these privacy issues partly arise from the system architecture architectures. For instance, centrality introduces a single point of failure, data leakage, and trust-related vulnerabilities. On the other hand, decentralized architecture introduces complexity and poor system control which may also lead to security compromises and general system inefficiencies [16]. A hybrid architecture has also been introduced to bridge the gap between centralized and decentralized architecture. However, despite these, the architecture-related challenges particularly system efficiency vis security and privacy vulnerabilities are still challenging.
- *Technology-based System Security and User Privacy Issues:* Similar to the Architecture-based issues, some security and privacy vulnerabilities arise from sensing technologies chosen for the design. For instance, BLE 4.0 and 4.1 have been discovered to be vulnerable to some attacks such as passive eavesdropping, man in the middle, relay, and denial of service attacks, etc. [152]. Similarly, GPS is known to be susceptible to identity leakage. Other sensing technologies also have various vulnerabilities. Therefore, research efforts are required to subject the various sensing technologies to more relevant security and privacy attacks to discover more vulnerabilities to guide against them.
- *Sensors’ accuracy issues:* The precision, accuracy, and efficiency of contact tracing and social distancing systems are fully dependent on the accuracy of the sensor utilized in the design. For instance, RF-based sensing devices such as BLE, WI-FI, GPS are affected by various factors such as multi-part propagation errors, environmental factors, reflection, refraction, and absorption [137]. Consequently, the sensing accuracy of such systems may not be fully guaranteed. Similarly, other devices such as sensors are limited by ranges and angles of coverage. Therefore, enhancing the precision, accuracy, and efficiency of contact tracing and social distancing systems is still an open research direction.
- *System Errors due to Implementation Platforms:* Smartphones are the most preferred implementation platform for contact tracing and social distancing systems because of their ubiquity. Other popular implementation platforms for contact tracing and social distancing systems include bracelets, bangles, facemask, etc. Nevertheless, smartphones and other devices are prone to sensing errors if positioned in a place where human muscle tissues or other obstacles can obstruct their signal line of sights or if positioned in different orientations or alignments such as putting them in back or chest pockets (for phones) or due to body movement in for other devices. Furthermore, being that some users place their smartphones inside other containers such as briefcases and handbags, it implies that readings taken from the smartphone’s sensors may not represent the accurate colocation distances [60,212]. Therefore, enhancing the system’s accuracy despite the various scenarios is still challenging.
- *System Sensing Errors Due to Proximity Estimation Technique*: Because BLE is the preferred sensing technology, the RSSI is the most popular proximity estimation technique for contact tracing and social distancing systems. However, RSSI accuracy is affected by changes in the environment. For instance, if a new infrastructure such as furniture, refrigerator, etc., are introduced to the same environment, or the distance estimation is performed in an entirely new location, the RSSI value changes due to the shadowing, shading effects, and multipath losses in the different environments [137]. Although several research efforts have been published to enhance RSSI accuracy, there is still room for further enhancements.
- *Incorporation of User Diagnostic Features:* Existing contact tracing and social distancing systems lack basic user diagnostic features such as temperature measurements, blood pressure and Blood oxygen level measurements, sneezing and coughing sensing, etc. These features if incorporated will enhance system effectiveness.
- *Improvement of System Adoption Rates:* It has been noted that contact tracing and social distancing apps perform more effectively if accepted by the general populace. With high adoption rates, flattening the infection curve is easy. However, existing contact tracing still suffers a low adoption rate. Therefore, researcher efforts are needed to come up with frameworks that will enhance contact tracing and social distancing systems adoption rate.
- *Application of IoTs to Achieve Privacy-Preserving Contact Tracing*: The security vulnerabilities of IoT devices pose a great challenge against its application in combating infectious diseases particularly in contact tracing and social distancing systems where security and User privacy are critical, achieving privacy-preserving contact tracing.
- *Application of SDN in Contact Tracing and Social Distancing Systems*: One major challenge of contact tracing and social distancing systems is getting the system architecture right. Therefore, in our opinion, since SDN is an emerging network architecture, its potential in this regard should be explored by the research community.
- *Application of 5G in Contact Tracing and Social Distancing System*: 5G is the network of the now designed for cross-domain applications. However, just like other generations of cellular networks, the accuracy in proximity estimation in a few meters (2 m for example) is a major challenge. Therefore, frameworks of achieving high precision proximity in a few meters (as required for contact tracing and social distancing) are still an open research issue.

## 6. Conclusions

The review work is an extensive study of articles published on the use of technologies as a non-clinical measure against COVID-19. The study specifically reviewed digital methods of contact tracing and social distancing schemes for combating the spread of coronavirus. In the study, contact tracing methods were reviewed under three sub-headings, namely, technologies, protocols, and apps. Our review of technologies in contact tracing systems shows that most contact tracing system developers implement Bluetooth low energy (BLE) as the preferred proximity sensing technology and smartphones as the implementation platforms. This is due to the availability of mobile phones as part of human lives and BLEs are already fitted in existing smartphones by the manufacturers. Other mobile phone-fitted sensors such as GPS and Wi-Fi are also sometimes used in contact tracing systems as proximity sensing technology. On the part of social distancing, the study identified five major approaches of achieving social distancing, namely, keeping a distance, crowd control, face mask detection, isolation/quarantine, and virtual interactions. Finally, we observed that a combination of contact tracing and social distancing schemes when properly implemented will go a long way to minimize the spread of contagious diseases. Finally, the study highlights key open research areas where interested researchers and experts can channel more efforts to enhance the effectiveness of contact tracing and social distancing methods as a non-pharmaceutical intervention against COVID-19.

## Figures and Tables

**Table 1:** Summary of recent literature on technology-based and non-pharmaceutical interventions against COVID-19.

References	The Objective of the Study	Limitations	Significance of Our Study	Research Gap
[11]	Review of various emerging technologies that contribute to the development of social distancing	Did not discuss the various protocols and apps deployed for social distancing	Provides a detailed review of apps, protocols, and technologies for both contact tracing and social distancing	Contact tracing schemes were not included in the study
[12]	To perform a systematic review on COVID-19 mobile apps for contact tracing	Focuses on Apps without discussing the technologies and the protocols	Provides a detailed review of apps, protocols, and technologies	Social distancing schemes were not included in the study
[13]	To analyze existing contact tracing protocols, apps, and underlying technologies to identify their strength and weakness	Did not perform in-debt analysis of the various proposed protocols	Provides a detailed review of apps, protocols, and technologies	Social distancing schemes were not included in the study
[14]	A comprehensive survey of contact tracing apps attributes	focused on the architecture of contact tracing apps and the respective cyber security vulnerabilities	Provides a detailed review of apps, protocols, and technologies for both contact tracing and social distancing systems	Social distancing schemes were not included in the study
[16]	To perform qualitative synthesis of digital contact tracing Systems	The focus is on the impact of adoption rate, proximity accuracy, and public’s trust on the effectiveness of digital contact tracing systems	Provides a detailed review of the various apps, protocols, and technologies	This study failed to incorporate social distancing schemes and contact tracing protocols in the review
[17]	To conduct a review of global deployment of contact tracing apps	The emphasis on Bluetooth based apps	Offers a comprehensive study of the various apps developed using different technologies	This study is limited to only single technology for contact tracing. No social distancing schemes in the review
[18]	To study the combination of Blockchain and advanced cryptography for security and privacy in the fight against COVID-19	Only analyzed recent Blockchain-based solutions for contact tracing and the management of immune/vaccine certifications	Not limited to Blockchain but Provides a detailed review of apps, protocols, and technologies for both contact tracing and social	Focused on Blockchain for contact tracing. No Social distancing schemes
[19]	Examines the extent to which design and implementation considerations for contact tracing apps are detailed in the available literature	Focused on design and implementation considerations of contact tracing systems	Provides a detailed review of apps, protocols, and technologies for both contact tracing and social	Social distancing schemes were not included in the study
[20]	To review big data analytics, artificial intelligence, and nature-inspired computing models for accurate detection of COVID-19 pandemic cases and contact tracing	Limited to artificial intelligence, nature-inspired computing, and big data analytic for epidemic detection and contact gracing	Provides a balanced study of the various technologies including artificial intelligence and computer vision methods for contact tracing and social distancing	Social distancing schemes were not included in the study
[21]	To review digital contact tracing apps	This study did not consider proximity accuracy in the review	Identified proximity accuracy issues in contact tracing systems	Social distancing schemes were not included in the study
[22]	To analyze existing contact tracing apps and examine their underlying technologies, public reception, and data management procedure	This study did not consider proximity accuracy in the review	Identified proximity accuracy issues in contact tracing systems	Social distancing schemes were not included in the study
[23]	To survey existing contact-tracing apps and organize them based on underlying technologies	Failed to consider the protocols employed in the surveyed apps	Provides a detailed review of apps, protocols, and technologies for both contact tracing and social distancing	Social distancing schemes were not included in the study
[24]	To analyze the opportunities and challenges of integrating emerging technologies into contact tracing systems	Focuses on emerging technologies without discussing the apps and the protocols	Provides a detailed review of apps, protocols, and technologies	Social distancing schemes were not included in the study
[25]	To provide the research community with new insights into the ways AI and big data can help in the fight against COVID-19	Did not specifically relate the study to contact tracing and social distancing	Provides a detailed review of apps, protocols, and technologies for both contact tracing and social distancing	Both contact tracing and social distancing schemes were not included in the study
[26]	To provide a review and discussion on the contribution of AI in the fight against COVID-19	Did not specifically relate the study to contact tracing and social distancing	Provides a detailed review of apps, protocols, and technologies for both contact tracing and social distancing	Both contact tracing and social distancing schemes were not included in the study

**Table 2:** Summary of Bluetooth-based contact tracing Apps.

Architecture	Apps	Protocols	Strengths	Weaknesses	References
Centralized	Tracetogether of Singapore	BlueTrace	Does not disclose user information	Suffers Single point of failure and drains user device battery	[33]
	Australian CovidSafe	BlueTrace	Does not disclose user information	Suffers Single point of failure and was hosted by a non-national company	[47]
	AB Tracetogether	BlueTrace	Does not disclose user information	Suffers single point of failure and does not work well in Apple iOS	[48]
	Aarogya Setu	Not specified	Transparent in privacy and security policies	Single point of failure and possibility of attacks through phone numbers	[50]
Decentralized	SwissCOVID	Apple-Google and the DP-3T	Not susceptible to single point of failure	Vulnerable to false positive and linkage attacks	[53]
	The German Corona-Warn-App	Apple-Google framework	The majority of the processes are performed by user devices	Still susceptible to single point of failure	[55]
	The NHS COVID-19 App of United Kingdom	Apple-Google framework	Integration of QR Code into the system	Vulnerable to user privacy leakage	[57]

**Table 3:** Summary of Bluetooth-based contact tracing Protocols.

Architecture	Protocols	Apps	Strengths	Weaknesses	References
Centralized	BlueTrace	Tracetogether of Singapore, Australian CovidSafe and AB Tracetogether	Does not disclose user information	Trust issues and Single point of failure	[33,47]
	Not specified	Aarogya Setu	Transparent in privacy and security policies	Single point of failure and possibility of attacks through phone numbers	[50]
Decentralized	Apple-Google and the DP-3T	SwissCOVID	Not susceptible to single point of failure	vulnerable to false positive and linkage attacks	[53]
	Apple-Google framework	The German Corona-Warn-App	The majority of the processes are performed by user devices	Still susceptible to single point of failure	[55]
		The NHS COVID-19 App of United Kingdom	Integration of QR Code into the system	Vulnerable to user privacy leakage	[57]
Hybrid	Contra Corona	nil	Balances centralized and decentralized architectures	Not implemented for public use	[43]
	Epione	nil	Balances centralized and decentralized architectures	Not implemented for public use	[44]
	Desire	nil	Balances centralized and decentralized architectures	Not implemented for public use	[45]

**Table 4:** Centralized GPS-based contact tracing apps.

Applications	Architecture	Strengths	Weaknesses	References
Philippine WeTrace	Centralized	Detects symptoms such as catarrh, cough, difficulty in breathing, and fever	Poorly developed, delays in loading and drains phone batteries.	[63]
South Corona-100 m	Centralized	Has notification features such as radius alerts and personal hygiene reminders	App abuses user privacy by displaying personal data	[64]

**Table 5:** Decentralized GPS-based contact tracing apps.

Applications	Architecture	Strengths	Weaknesses	References
Israeli Hamagen	Decentralized	The app is partially distributed	Suffers poor accuracy and is not suitable for indoor application.	[60,61]
The Iranian AC-19	Decentralized	User self-diagnostic feature	Government tracks users via collected sensitive data	[65,66]
The USA private Kit: Safepaths	Decentralized	Open source and supports user privacy preservation	Likelihood of tracking user since the app keeps a record of user location	[67]
The Pakistan COVID-19 PK	Decentralized	Has notification features such as radius alerts and personal hygiene reminders	Serves only as an infection notice board	[68]

**Table 6:** AI-based contact tracing apps.

Applications	Architecture	Strengths	Weaknesses	References
The ChineseAlipay and WeChat mobile app	hybrid	The integration of smart wristwatch makes the system more effective	Unavailability of training data makes the systems ineffective	[77]
The LeaveHomeSafe of Hong Kong	decentralized	The system is designed not to disclose user personal information	Users can easily be linked to the QR code	[78]

**Table 7:** Contact tracing Protocols using other technologies.

Technologies	Descriptions	Strengths	Weaknesses	References
Wireless Fidelity (Wi-Fi)	IEEE 802.11 standard-based communication technology that interconnects devices without the use of cables	Low deployment cost	Poor proximity estimation accuracy	[81,82,83,84]
Smartphone Magnetometer Traces	A concept that smartphones maintain high linear correlation in their magnetometer traces if positioned at close range	Does not reveal device identities	Magnetometer traces suffer distortion in a ferromagnetic obstacle and require a high energy requirement	[60,61,89]
Cellular Network (CN)	technologies used for mobile phone communication	High coverage area	Poor proximity estimation accuracy	[91,95]
Radio Frequency Identifier (RFID)	Real-time location systems that use unique codes for automatic and contactless objects identification	does not depend on line of sight for its operations	RFID tags are limited in storage, hence may not accommodate many security algorithms	[104]
Internet of Things (IoT)	Technologies for detection of COVID-19 symptoms	Adds intelligence to devices	Security vulnerabilities	[115,116]
Near Field Communication (NFC)	wireless communication protocol for objects at close range of less than 4 cm at about 424 kbps	provides a seamless, fast, and reliable platform for device communication and data exchange	Suitable for objects at about 4cm range and not for higher ranges	[111]
Blockchain	Distributed database for data management	No central control over the database	The technology is relatively new, requires high technical skills, and high installation cost	[90]
Software-Defined Networking (SDN)	Employs standardized network API for network configuration, data storage, and data sharing	Eliminates vendor-specific dominance and control	Requires highly skilled personnel to implement	[122,123,124,128,129,130]

**Table 8:** Individual-centric approach to keeping a distance.

Sensing Devices	Platforms	Strengths	Weaknesses	References
BLE	Android-based Smartphone	The system performs both proximity detection and developed risk optimization plan	At 95% of CVaR confidence, the model can handle only 45.11% of the risks associated with the user’s safe distance estimation	[176]
BLE	Android-based Smartphone	Notification is by real-time popups on a phone screen	The system android specific. It does not accommodate users of other operating systems such as IOS	[177]
BLE	Smartphone	The system has an inbuilt machine learning algorithm that determines if social distancing is bridged or otherwise	The study was limited to only Xiaomi Redmi 7A and Bluetooth Huawei CAM-L03	[178]
Oscillating magnetic field sensor	Wearable device	Proposed system not prone to multipath propagation errors seen in wireless technologies	The prototype needs to be miniaturized	[109]
Passive infrared sensors	Wearable cap	The system achieved 360° coverage using 3 PIRs	A shift in the alignment of any of the PIR sensors reduces the sensing coverage of the system	[182]
Ultrasonic sensor (US)	Wearable ID card	Automatic alarm notification	Ultrasonic has poor sensing coverage and could detect inanimate objects which could lead to errors	[183]
Ultrasonic sensor (US)	Wearable device with LCD component	Automatic notification	Sensing coverage and could detect inanimate objects which could lead to errors	[184]
Hybrid of BLE and UWB	Smartphone	Proposed solution interoperable with other BLE or UWB-based solutions	UWB radios increase deployment cost and complexity	[186]
Hybrid of BLE and ultrasound	Smartphone	Real-time notification in a decentralized approach	Ultrasound-based systems are costly and complex to deploy	[187]

**Table 9:** Location-based social distancing using computer vision.

Video Sources	Deep Learning Object Detection Techniques	Distance Estimation Technique	Object Detection Results	Roles	Strength	Weaknesses	Ref.
CCTV	YOLO v4	Inverse perspective geometric mapping (IPM)	99.8%	enforcing physical distance	The combination of MS COCO and Google Open Image datasets enhances detection accuracy	Model is complex	[193]
Fixed monocular camera	Faster R-CNN and YOLOv4	Euclidean distance Formula	About 42% for R-CNN and 43% for YOLOV4	Keeping a distance and entry regulation	The framework is user privacy oriented	Poor pedestrian detection	[194]
Single motionless time of flight (ToF) camera	YOLOV4	Time of Flight	98.74%	enforcing physical distance	The mobility feature of the framework makes it unique	The system was developed for a single motionless ToF camera and not for various kinds of cameras	[195]
CCTV	YOLO v3	Pair-wise vectorized approach	84.6%	Keeping a distance	The trials using other object detection models: Faster RCNN, SSD, and YOLO v3 further validate the approach	Detection accuracy needs enhancement	[197]

**Table 10:** Crowd regulation measures.

Measures	Technologies	Mode of Operation	Strengths	Weaknesses	Ref.
Real-time monitoring	Wi-Fi, Sensors, IoT, computer vision	Monitors occupants in a location and sends alarms if a threshold is exceeded or if social distancing rule is violated	The is an enforcement measure that is difficult to bye-pass	Possibility of poor sensing accuracy	[199,200,201,202,204,206]
Scheduling	Blockchain	Arranges people both service personnel and recipients of the service in a manner that does not constitute a crowd	This measure is preventive in its approach and is easy to implement	Makes services available to only the scheduled persons which might inconvenience others	[207,208,209,210]
Incentives	Blockchain	Provides compensation and another lobbying to people who obey social distancing rules	This measure is humanitarian in its approach, hence can ease people’s burden during pandemic periods	Might require high cost	[211]

**Table 11:** Virtual interaction platforms for social distancing.

Virtual Platforms	Mode of Operation	Strengths	Weaknesses	References
Social media	Provides a medium for people to interact and share information and multimedia data	Information dissemination is rapid and easily accessible	Not regulated in many countries hence could spread false information	[220,221,222,223,224,225]
Online interactive system	A platform for remote interactions especially for business and academic purposes	Cost saving as distance is not a barrier for participation	Requires stable internet for seamless streaming	[226,227,228,229]
Online shopping and robotic delivery	A medium through which sales and purchases can be made virtually and probably delivered via robotic services	Time saving and eliminates inconveniences of going to physical shops	May incur additional cost	[190,230,231,232,233,234,235,236]
Augmented and virtual realities	Launches user into a virtual environment or adds some audio-visual enhancement to a user	Creates artificial environment or enhances existing environment to give the user a new understanding	High cost of acquisition	[237,238,239,240,241,242,243]

**Table 12:** Facemask detection technologies.

Mask Detection Techniques	Data Set	Detection Accuracy	Strengths	Weaknesses	References
Combination of Different super-resolution and classification networks (SRCNet) for images	3835 medical masks dataset images wearing, 671 no mask-wearing, 134 incorrect wearing, and 3030 correct wearing	98.70%	The use of super-resolution and classification networks (SRCNet) in the framework is novel and relatively efficient	The Mask identification period is 10 images per second which is below the video frame rate of 24 images per second. No video in a dataset	[245]
Facemasknet which uses Deep Learning	35 images of 10 wearing mask, 10 no mask and 15 incorrect wearing of a mask	98.6%	The framework works for both still images and video streams	Inadequate training data. Could not classify images of partially hidden faces or images higher than 10 feet	[246]
CNN in combination with Keras/TensorFlow and OpenCV	1376 images of 690 with mask, 686 no mask. 560 later selected—80% training and 20% testing data	98.86% with training data and 96.19% with test data all together above 96%	The trial was conducted using a reasonable number of dataset	Delays in image detection time	[247]
Retinafacemask with ResNet or MobileNet backbone, FPN, and context attention modules	7959 images being a total for both with or without a face mask	2.3% and 1.5% > baseline accuracy in face and mask respectively	The trial was conducted using a reasonable number of the dataset which enhances detection accuracy	Some components of the framework do not work well with ResNet	[248]
SSDMNV2 which employs OpenCV deep neural networks with MobilenetV2 image classifier	5521 images for both with or without a mask	92.64%	The trial was conducted using a reasonable number of the dataset which enhances detection accuracy	The study could not evaluate the framework in a Real-world application scenario	[249]
Pynq-YOLO-Net—lightweight Convolutional Neural Network (CNN) and the YOLO object detection framework	Very large dataset—from RMFD, MFDD, SMFRD, and MAFA	97%	A very large dataset that enhances detection accuracy	The framework was not evaluated on video surveillance systems nor tested on real conditions	[250]
YOLO v2 based ResNet-50 model	1415 images being filtered images from MMDS and FMD	81%	Since the study focused on medical face mask detection, it will be useful in the hospital setting where medical facemasks are mostly used	The framework adopted classical machine learning methods which have slow detection time and low accuracy	[251]
InceptionV3 with image augmentation	1570 simulated masked face dataset of masked and 785 unmasked	100%	The framework integrated image augmentation techniques to enhance performance	The proposal failed to classify the type of mask	[252]

## Data Availability

Not applicable.

## Abbreviations

The following abbreviations were used in the paper:

ACO	Ant Colony Optimization
AI	Artificial Intelligence
AP	Access Point
AR	Augmented Reality
AWS	Amazon Web Services
BBR	Bluetooth Basic Rate
BC	BlockChain
BLE	Bluetooth low energy
CCTV	Closed Circuit Television
CAUDHT	Contact tracing Application Using a Distributed Hash Table
CN	Cellular Network
CNN	Convolutional Neural Networks
DP-3T	Distributed Privacy-preserving proximity tracing
DSS	Decisional Support System
ECDH	Elliptic Curve Diffie-Hellman
EDR	Enhanced Data Rate
ENACT	Encounter-based Architecture for Contact Tracing
FHSS	Frequency Hopping Spread Spectrum
FMD	Face Masked Dataset
FPN	Feature Pyramid Network
GDP	Gross Domestic Product
GDP	General Data Protection Regulation
GPS	Global Positioning System
IoT	Internet of Thing
IPM	Inverse Perspective Mapping
ISM	Industrial Scientific Medical
ITC	Information Technology and Communications
LIDAR	Light Detection and Ranging
MAC	Media Access Control
MERS	Middle East Respiratory Syndrome
MILP	Mixed Integer Linear Programming
MIOU	Mean Intersection Over Union
MSC	Mobile Switching Center
MMD	Medical Mast Dataset
MS COCO	Microsoft Common Objects in Context
NHS	National Health Service
NFC	Near-Field Communication
NIST	National Institute of Standards and Technology
NMT	Nomadic Mobile Telephone
NTP	Network Time Protocol
PACT	Privacy-Sensitive Protocol and Mechanism for Mobile Contact Tracing
PBC	Public BlockChain
PBN	Public BlockChain Network
PEPIN-PT	Pan-European Privacy-Preserving Proximity Tracing
PIR	Passive Infrared Sensors
PHY	Physical Layers Extensions
POC	Proof of Consensus
PoW	Proof of Work
PSO	Particle Swarm Optimization
PPC	Privacy-Preserving COVID-19 Contact Tracing App
QR	Quick Response
R-CNN	Region-based Convolutional Neural Networks
RF-CNN	Recurrent Fuzzy Coupled Cellular Neural Network
RSSI	Received Signal Strength Indicator
SARS	Severe Acute Respiratory Syndrome
SIG	Special Interest Group
DVD	Simulated Masked Face Dataset
SORT	Simple Online Real-Time Tracking
SRCNet	Super-Resolution and Classification Networks
SSD	Single Shot Detector
SSID	Service Set Identifier
STK	Short Term Keys (STK)
TCN	Temporary Contact Number
TDOA	Time Differential of Arrival
TOA	Time of Arrival
Wi-Fi	Wireless Fidelity
WHO	World Health Organization
RFID	Radio Frequency Identifier
RNN	recurrent neural network
WEP	Wireless Equivalent Privacy
UWB	Ultra-Wide Band
VR	Virtual Reality
